# ICOS regulates IL-10 production in group 2 innate lymphoid cells via cholesterol and cortisol biosynthesis

**DOI:** 10.1172/JCI193134

**Published:** 2025-07-08

**Authors:** Yoshihiro Sakano, Kei Sakano, Benjamin P. Hurrell, Mohammad H. Kazemi, Xin Li, Stephen Shen, Omid Akbari

**Affiliations:** Department of Molecular Microbiology and Immunology, Keck School of Medicine, University of Southern California, Los Angeles, California, USA.

**Keywords:** Immunology, Pulmonology, Allergy, Asthma, Cholesterol

## Abstract

Group 2 innate lymphoid cells (ILC2s) play a crucial role in inducing type 2 inflammation in the lungs in response to allergens. Our study investigated the regulatory mechanism of IL-10 production by ILC2s and its impact on airway hyperreactivity (AHR), focusing on the role of ICOS. We found that inhibiting ICOS in pulmonary ILC2s significantly enhanced IL-10 production. The absence of ICOS reprogrammed ILC2 steroid metabolism, leading to increased cholesterol and cortisol biosynthesis and subsequent glucocorticoid receptor (GR) activation. This reprogramming regulated MAF and NFIL3 activation, promoting IL-10 production. Notably, in vivo GR inhibition or ILC2-specific GR deficiency exacerbated AHR development in multiple mouse models. We extended these findings to human ILC2s, demonstrating concordant results between murine models and human cells. Our results indicate that ICOS negatively regulates IL-10 production in ILC2s by controlling cholesterol and cortisol biosynthesis. This mechanism provides new insights into the complex interplay between ILC2s, ICOS, and glucocorticoid signaling in the context of allergic airway inflammation.

## Abstract 2

ICOS in the lung group 2 innate lymphoid cells (ILC2) regulates IL-10 production by remodeling cholesterol metabolism, suppressing glucocorticoid receptor and controlling MAF and NFIL3 activity. These findings may contribute to novel therapeutic strategies to promote IL-10 production by ILC2s.

## Graphical abstract

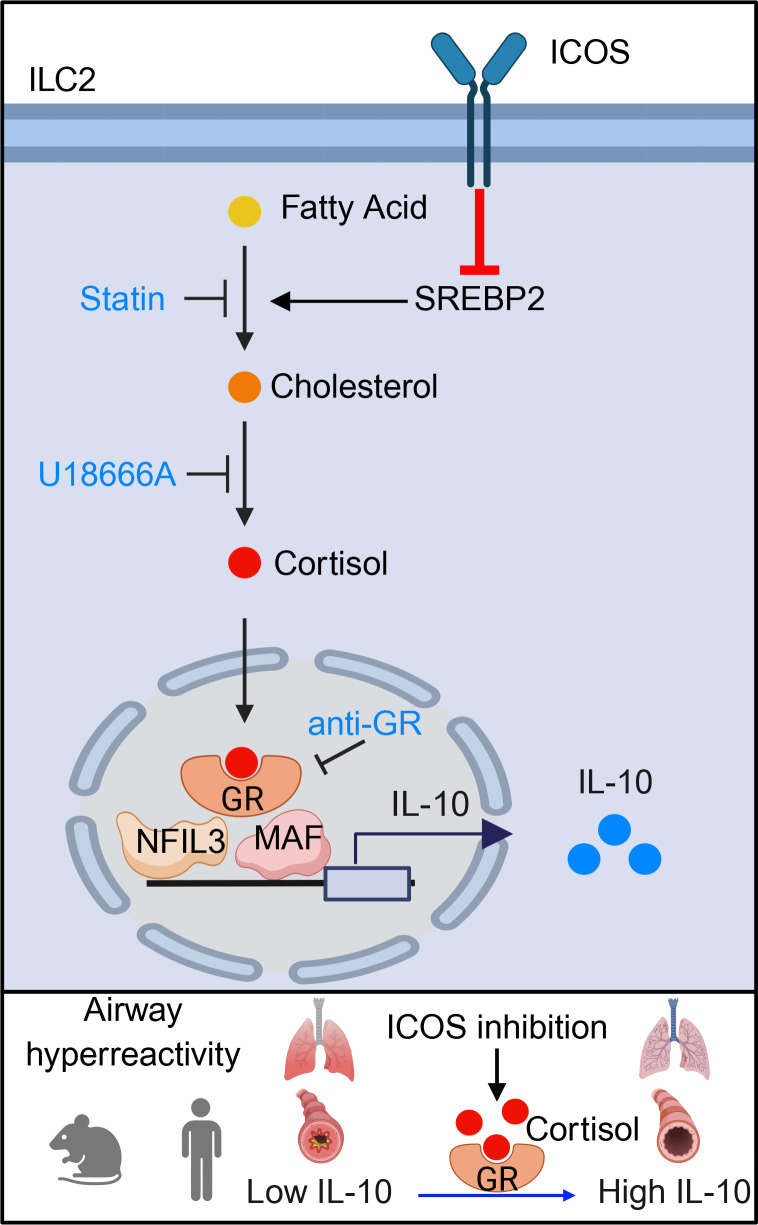

## Introduction

Asthma is a chronic inflammatory disease of the airways, characterized by persistent airway inflammation, bronchoconstriction, and airway hyperreactivity (AHR). The disease affects over 300 million individuals globally with a prevalence exceeding 3,000 per 100,000 people and a continuing upward trend (1). Although current treatment modalities, including corticosteroids, anti-inflammatory agents, and β-adrenergic agonists, show partial efficacy, higher rates of resistant patients necessitate developing novel therapeutics (2). Allergic asthma is primarily driven by type 2 cytokines, such as IL-4, IL-5, IL-9, and IL-13 (3). Conversely, IL-10, an anti-inflammatory cytokine that suppresses type 2 immune responses, has emerged as a potential therapeutic target (4). Notably, asthmatic patients exhibit reduced IL-10 production compared with healthy individuals, suggesting that therapies aimed at enhancing IL-10 in the lungs may provide a novel approach to controlling allergic asthma (5).

Recent studies have highlighted the complex role of group 2 innate lymphoid cells (ILC2s) in the pathogenesis and exacerbation of allergic asthma. These tissue-resident innate immune cells, characterized by their production of type 2 cytokines, have become key targets for novel therapeutic interventions (6). While initial strategies focused on broadly suppressing ILC2 activation showed promise, accumulating evidence has revealed a more nuanced understanding of ILC2 biology (7–10). Studies from our group and others have identified distinct ILC2 subpopulations with divergent functional profiles, including pro-inflammatory cytokine-producing subsets and others that secrete anti-inflammatory mediators such as IL-10 (11–13). This functional plasticity, regulated by alterations in intracellular metabolism, and associated transcription factor expression, suggest a paradigm shift in ILC2-targeted therapies for severe asthma (7, 9, 12). Moving beyond general suppression of ILC2s, a more refined approach that selectively inhibits pro-inflammatory ILC2 subsets while promoting the activation of anti-inflammatory, IL-10–producing ILC2s may offer a more effective strategy for modulating inflammation. The strategy employed in this study focuses on exploiting the innate biology and plasticity of ILC2s. The potential benefits of this approach extend beyond improving therapeutic outcomes for patients with severe asthma, encompassing the enhancement of lung tissue homeostasis.

ICOS is a critical molecule in the immune system, playing a multifaceted role in T cell activation and immune regulation (14, 15). Expressed on activated T cells, ICOS interacts with its ligand (ICOSL) on antigen-presenting cells to deliver costimulatory signals (16). Recent studies by our group and others have revealed that ICOS and ICOSL are also expressed on ILC2s, where they promote inflammatory responses by enhancing cell proliferation and the production of type 2 cytokines (17, 18). These findings underscore the importance of ICOS in both adaptive and innate immune responses. In T cells, recent studies have elucidated a key role for ICOSL, expressed on mature dendritic cells, in promoting the differentiation of naive CD4^+^ cells into IL-10–producing Treg cells (19). Additionally, ICOS–ICOSL interactions have been shown to stimulate IL-10 production in Tregs (14). However, the relationship between ICOS and IL-10 production in ILC2s remains poorly understood. Given the complex role of ILC2s in asthma pathogenesis and the potential for therapeutic targeting of these cells, investigating the relationship between ICOS and IL-10 production in ILC2s could provide valuable insights into immune regulation and lead to novel therapeutic approaches for severe asthma. Therefore, this study aims to investigate the mechanisms linking ICOS signaling to IL-10 production in ILC2s in murine allergic models, with the goal of uncovering new targets for the treatment of severe asthma and other ILC2-mediated inflammatory conditions.

Intracellular metabolism is frequently implicated as a regulatory factor in cytokine production by immune cells (20). In previous studies, we demonstrated that cytokine production is also regulated by changes in intracellular metabolism in ILC2s (7, 9, 21). Recent reports have suggested that cholesterol biosynthesis plays a role in cytokine production (22). However, there is a limited amount of information on the role of cholesterol biosynthesis in ILC2s in relation to cytokine production. Consequently, this study was initiated to examine whether cholesterol biosynthesis is involved in IL-10 production in ILC2s.

Cortisol, a hormone produced from cholesterol, is well known for its role in regulating inflammation (23). For example, cortisol has been shown to inhibit the activation of M1 macrophages, a type of inflammatory cell, and promote the differentiation of M2 macrophages, which are anti-inflammatory (24, 25). Additionally, cortisol has been observed to induce IL-10 secretion in M2 macrophages (24). Given these findings, cortisol may be closely related to IL-10 production. Therefore, this study also aims to investigate whether cortisol exerts a similar effect on IL-10 production in ILC2s.

Using ICOS-deficient models, we found that ICOS not only promotes type 2 cytokine production, but also inhibits IL-10 synthesis in ILC2s. Mechanistically, our results demonstrate that ICOS regulates cholesterol biosynthesis and influences cortisol production in ILC2s, thereby modulating key transcription factors such as MAF and nuclear factor, interleukin 3 regulated (NFIL3). These transcription factors, in turn, regulate IL-10 production. Notably, the glucocorticoid receptor (GR) plays a central role in this process, as ILC2-specific loss of GR markedly reduced IL-10 production in both ex vivo and in vivo settings, leading to exacerbated AHR. To further substantiate the involvement of GR in ICOS signaling, we demonstrate that ILC2-induced AHR was exacerbated following GR antibody blocking in ICOS-KO mice. Similar results were observed in human ILC2s (hILC2s), confirming that inhibition of ICOS signaling promotes IL-10 production and alters the expression of genes involved in cortisol biosynthesis. Furthermore, inhibiting cholesterol biosynthesis and cortisol signaling led to reduced IL-10 production. In summary, our results show that ICOS regulates IL-10 production by controlling cholesterol biosynthesis and cortisol production in ILC2s. This discovery highlights a novel mechanism for regulating ILC2-mediated inflammation and paves the way for targeted therapeutic strategies for ILC2-dependent AHR.

## Results

### ICOS deficiency induces IL-10 secretion in ILC2s and modulates AHR.

**Figure 1:**
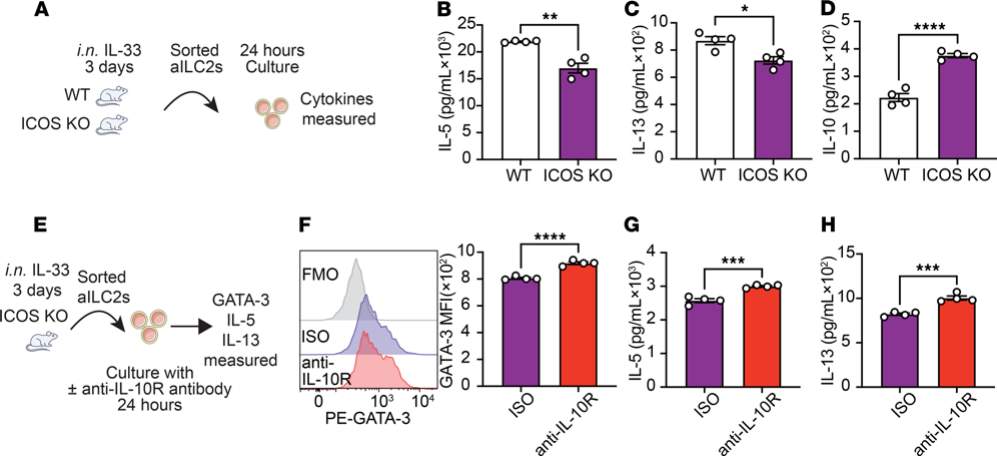
ICOS mediates IL-10 production from ILC2s. (**A**–**D**) WT and ICOS-KO mice received i.n. doses of rmIL-33 over 3 consecutive days. Lung ILC2s were isolated on day 4 and cultured. (**B**–**D**) Levels of IL-5 (**B**), IL-13 (**C**), and IL-10 (**D**) production in the culture supernatant were measured. *n* = 4. (**E**–**H**) Cohorts of ICOS-KO mice were i.n. challenged with rmIL-33 over 3 consecutive days. On day 4, lung ILC2s were isolated and cultured with isotype control (ISO) or anti–IL-10R antibody. (**F**) Representative plots of GATA-3 expression in ISO and anti–IL-10R groups and corresponding quantitation presented as GATA-3 MFI. *n* = 4. (**G** and **H**) Levels of IL-5 (**G**) and IL-13 (**H**) production in the culture supernatant were measured. *n* = 4. Data are presented as mean ± SEM and are representative of 4 independent experiments. Two-tailed Student’s *t* test was employed for statistical analysis; *P < 0.05, **P < 0.01, ***P < 0.001, and ****P < 0.0001. Schematic images were created in Adobe Illustrator. FMO, fluorescence minus one.

**Figure 2:**
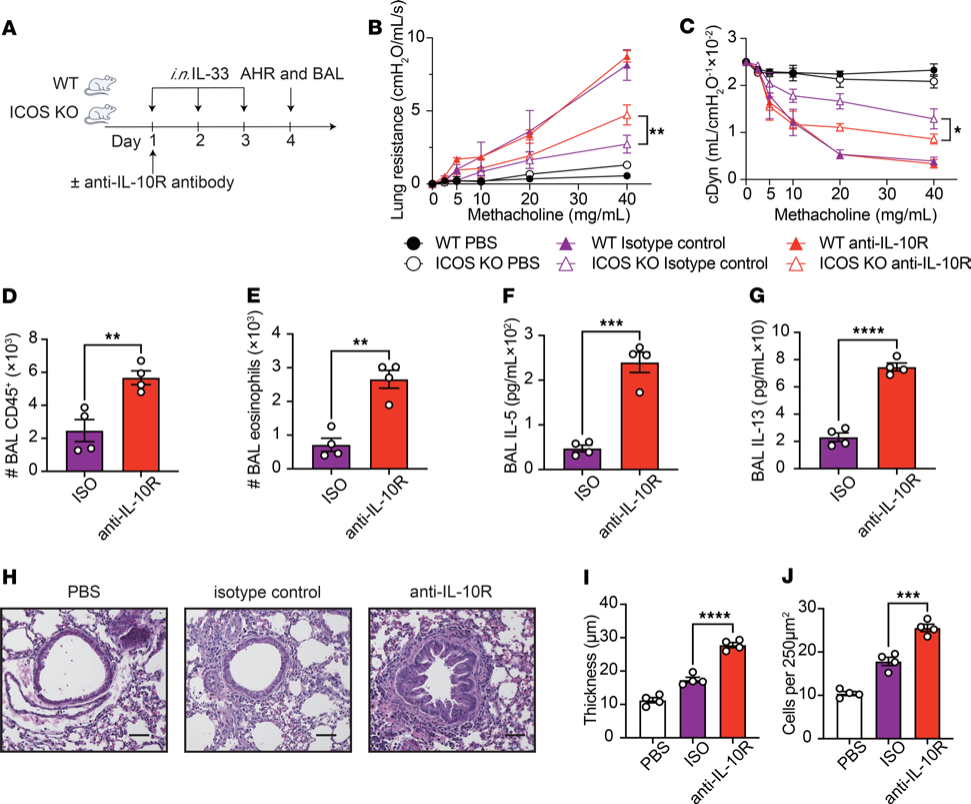
IL-10 affects the AHR. (**A**–**J**) WT mice received i.p. injections of isotype control or anti–IL-10R antibody (200 μg) on day 1 and were i.n. exposed to 0.5 μg of rmIL-33 or PBS for 3 days. Lung function and inflammation were assessed on the day 4. (**B** and **C**) Lung resistance (**B**) and dynamic compliance (**C**) in response to elevating doses of methacholine. *n* = 4. (**D** and **E**) The total number of CD45^+^ cells (**D**) and CD45^+^, Gr-1^–^, CD11c^–^, and SiglecF^+^ eosinophils (**E**) in BAL fluid are demonstrated in bar graphs. *n* = 4. (**F** and **G**) Levels of IL-5 (**F**) and IL-13 (**G**) in the BAL fluid are shown in bar graphs. *n* = 4. (**H**) Lung histologic sections stained with H&E are presented. Scale bars: 50 μm. (**I** and **J**) Quantification of airway epithelium thickness (**I**) and infiltrating cells (**J**). *n* = 4. Data are presented as mean ± SD or SEM and are representative of 3 independent experiments. Two-tailed Student’s *t* test or 1-way ANOVA followed by Tukey’s post hoc tests was employed for statistical analysis; *P < 0.05, **P < 0.01, ***P < 0.001, and ****P < 0.0001. Schematic images were created in Adobe Illustrator.

This study aimed to determine the effects of ICOS on ILC2 effector function. To this end, we administered IL-33 i.n. to WT and ICOS-KO mice for 3 days, followed by lung digestion and ILC2 isolation via cell sorting on the fourth day (Figure 1A). Lung ILC2s were characterized as CD45^+^, lineage^–^, CD127^+^, and ST2^+^ cells (Supplemental Figure 1A; supplemental material available online with this article; https://doi.org/10.1172/JCI193134DS1). Isolated ILC2s were then cultured for 24 hours, and the concentrations of various cytokines were measured in the culture supernatants by LEGENDplex (BioLegend). We found that ICOS-KO ILC2s produced significantly less type 2 cytokines IL-5 and IL-13 (Figure 1, B and C, and Supplemental Figure 1, B and C), while the production of IL-10, an anti-inflammatory cytokine, was surprisingly increased compared with WT ILC2s (Figure 1D and Supplemental Figure 1D). Furthermore, similar results were obtained utilizing intracellular staining (Supplemental Figure 1, E–G). To assess the impact of secreted IL-10 on ILC2 function, pure populations of activated lung ILC2s isolated from ICOS-KO mice were cultured with isotype control or anti–IL-10R blocking antibody, the receptor for IL-10, for 24 hours (Figure 1E). We found that IL-10R–blocked ILC2s exhibited a significant upregulation in GATA binding protein 3 (GATA-3) expression, a hallmark activation marker for ILC2s (Figure 1F and Supplemental Figure 2A). This observation was accompanied by an enhancement in the production capacity of IL-5 and IL-13 ex vivo (Figure 1, G and H, and Supplemental Figure 2, B and C). In an ILC2-dependent AHR model induced by *Alternaria alternata*, treatment with anti–IL-10R antibody (200 μg/mice) resulted in a significant upregulation of GATA-3 expression compared with the isotype control group (Supplemental Figure 2, D and E). This finding was consistent with the results observed in the ex vivo experiments. To investigate the effects of IL-10 produced by ILC2s in vivo on the development of AHR, an ILC2-driven AHR model was utilized. WT and ICOS-KO mice were challenged i.n. with IL-33 for 3 consecutive days, concurrently with i.p. administration of anti–IL-10R antibody (200 μg/mice) or isotype control on the first day of the challenge. On day 4, lung resistance and dynamic compliance were measured, bronchoalveolar lavage (BAL) fluid was collected and analyzed, and lung histology was examined to assess lung inflammation (Figure 2A). A comparison of PBS-treated mice with those challenged with IL-33 revealed worsened lung resistance and dynamic compliance in IL-33–challenged mice in response to increased doses of methacholine, a bronchoconstrictor. We notably found that ICOS-KO mice exhibited markedly diminished pulmonary resistance and higher dynamic compliance in comparison with their WT counterparts, confirming our previous findings (17). Conversely, ICOS-KO mice treated with anti–IL-10R antibody exhibited significantly increased lung resistance (Figure 2B) and lower dynamic compliance (Figure 2C) compared with isotype-treated mice. This finding is corroborated by the observation that the anti–IL-10R antibody–treated group exhibited a substantial increase in lung inflammation, as indicated by elevated CD45^+^ cells reflecting the total number of immune cells in the BAL fluid (Figure 2D and Supplemental Figure 2F) and eosinophils in the BAL fluid (Figure 2E and Supplemental Figure 2F). Furthermore, IL-5 (Figure 2F) and IL-13 levels (Figure 2G) in the BAL fluid were significantly elevated in the anti–IL-10R antibody–treated group compared with the isotype control group. Histological analysis of lung tissue (Figure 2H) further corroborated these results, with significantly increased epithelial thickness, a common manifestation of remodeling during airway inflammation (Figure 2I), and inflammatory cell counts, a common measurement of the extent of inflammation (Figure 2J), in the anti–IL-10R antibody–treated group compared with the isotype control group. Together, our results suggest that ICOS deficiency promotes IL-10 production in ILC2s and that IL-10 plays a role in regulation of ILC2-induced AHR and lung inflammation in ICOS-KO mice.

### ICOS signaling limits IL-10 production by regulating MAF and NFIL3 expression in ILC2s.

**Figure 3:**
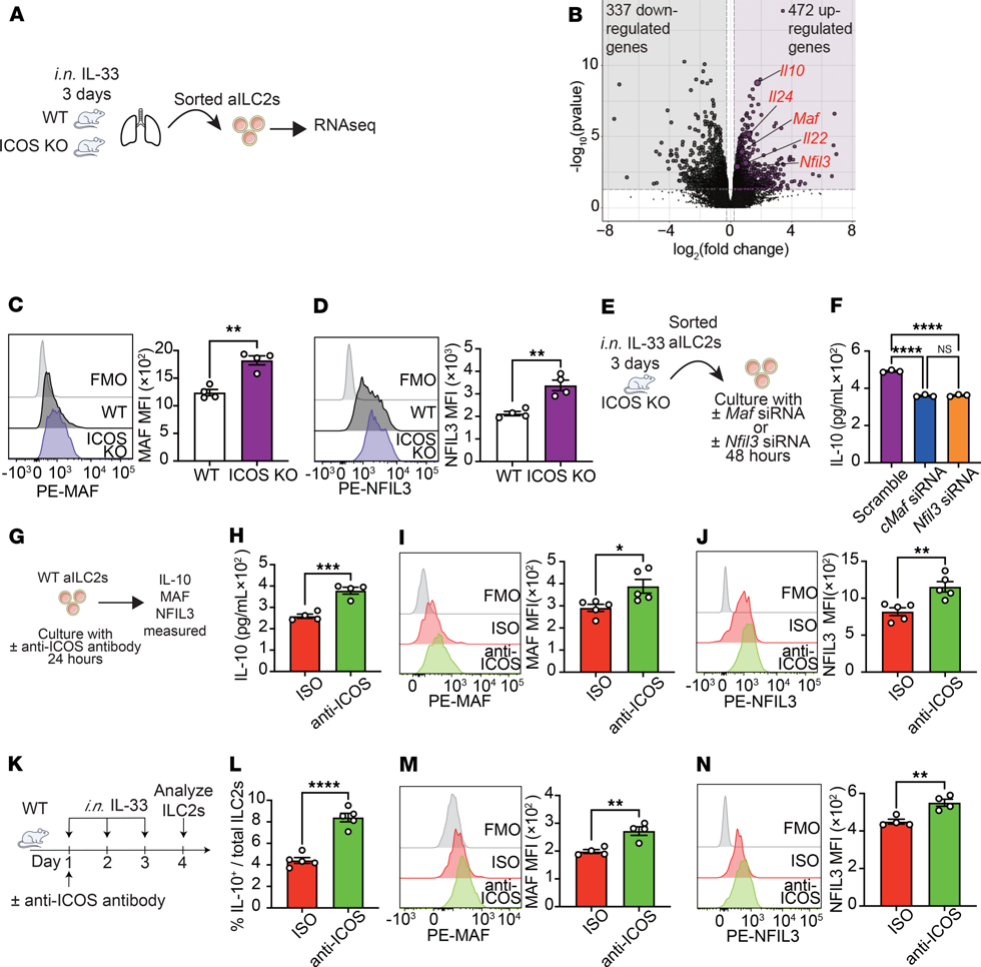
NFIL3 and MAF regulate IL-10 production via ICOS in ILC2s. (**A** and **B**) Pulmonary aILC2s were isolated from WT and ICOS-KO mice subjected to i.n. IL-33 challenges for 3 days. Subsequently, RNA-Seq was performed on ILC2s following 18-hour culture. (**B**) Total RNA from WT and ICOS-KO mice was extracted to perform a bulk transcriptomic analysis. Volcano plots represent differentially expressed genes. (**C** and **D**) Representative plots of MAF (**C**) and NFIL3 (**D**) expression levels in WT and ICOS-KO ILC2s are shown. Corresponding quantitation is presented as MFI. *n* = 4. (**E** and **F**) aILC2s from ICOS-KO mice were cultured with or without *Maf* siRNA or *Nfil3* siRNA. (**F**) Levels of IL-10 in the culture supernatant were measured. *n* = 3. (**G**–**J**) WT aILC2s were cultured with isotype control or anti-ICOS antibody. (**H**) Levels of IL-10 in the culture supernatant were measured. *n* = 4. (**I** and **J**) Representative plots of MAF (**I**) and NFIL3 (**J**) expression levels in each group are shown. Corresponding quantitation is presented as MFI. *n* = 4. (**K**–**N**) WT mice received i.p. injections of 500 μg anti-ICOS antibody or isotype control (ISO) on day 1 and were i.n. exposed to 0.5 μg of rmIL-33 or PBS for 3 days. On day 4, mice were euthanized. (**L**) Frequency of IL-10^+^ ILC2s in each group. *n* = 4. (**M** and **N**) Representative plots of MAF (**M**) and NFIL3 (**N**) expression levels in each group are shown. Corresponding quantitation is presented as MFI. *n* = 4. Data are presented as mean ± SEM and are representative of 4 independent experiments. Two-tailed Student’s *t* test or 1-way ANOVA followed by Tukey’s post hoc tests was employed for statistical analysis; *P < 0.05, **P < 0.01, ***P < 0.001, and ****P < 0.0001. Schematic images were created in Adobe Illustrator. FMO, fluorescence minus one.

We next isolated activated ILC2s from the lungs of WT and ICOS-KO mice and performed a transcriptomic analysis to investigate how ICOS regulates IL-10 production in these cells. We first isolated pulmonary active ILC2s (aILC2s) from WT and ICOS-KO mice subjected to i.n. IL-33 challenge for 3 days. Subsequently, we performed RNA sequencing (RNA-Seq) on ILC2s following 18-hour culture (Figure 3A). We found 809 differentially expressed genes between WT and ICOS-KO (337 downregulated genes and 472 upregulated genes) (Figure 3B). Furthermore, the expression of *Il22* and *Il24*, which are members of the IL-10 superfamily (26) as well as *Maf* and *Nfil3*, known to induce IL-10 (12, 27), were also significantly increased (Figure 3B). In support of our transcriptomic analysis, we confirmed the expression and upregulation of MAF and NFIL3 in ICOS-KO ILC2s compared with WT ILC2s at the protein level (Figure 3, C and D). To further support these findings, analysis using the *Alternaria* stimulation model revealed a significant increase in the expression of MAF and NFIL3 in ICOS-KO ILC2s (Supplemental Figure 3, A–C). We next investigated the role of ICOS, MAF, and/or NFIL3 in the regulation of IL-10 production. Activated lung ILC2s were isolated from ICOS-KO mice and cultured with *Maf* siRNA, *Nfil3* siRNA, or Scramble siRNA for 48 hours (Figure 3E). As expected, the expression of MAF in ILC2s cultured with *Maf* siRNA (Supplemental Figure 3D) and NFIL3 in ILC2s cultured with *Nfil3* siRNA (Supplemental Figure 3E) was significantly reduced compared with Scramble siRNA. Interestingly, however, ILC2s with reduced MAF and NFIL3 expression exhibited significantly lower IL-10 levels in the culture medium compared with Scramble siRNA (Figure 3F). To confirm the effects of ICOS on MAF, NFIL3, and IL-10 production, we next cultured pure populations of activated WT ILC2s with an anti-ICOS antibody or isotype control (Figure 3G). In confirmation of our findings using ICOS-KO mice, the levels of IL-10 in culture supernatants were significantly increased in WT ILC2s treated with an anti-ICOS antibody (Figure 3H). Similarly, the expression of MAF (Figure 3I) and NFIL3 (Figure 3J) was also increased in ILC2s treated with anti-ICOS antibody compared with controls. Consistent with these findings, analysis of the *Alternaria* stimulation model demonstrated a significant upregulation of MAF and NFIL3 expression in ILC2s following anti-ICOS antibody treatment (Supplemental Figure 3, F–H). Finally, we validated these findings in vivo using a mouse model of IL33-mediated airway inflammation. Cohorts of WT mice were challenged with IL-33 i.n. on days 1–3, concurrently with anti-ICOS antibody (500 μg/mouse) or an isotype control i.p. on day 1 (Figure 3K). Consistent with our previous findings, the frequency of IL-10^+^ ILC2s in lung ILC2s was significantly elevated in the anti-ICOS antibody–treated group compared with controls (Figure 3L). Furthermore, the expression of MAF (Figure 3M) and NFIL3 (Figure 3N) in ILC2s was also elevated in anti-ICOS antibody–treated mice compared with controls. To support these ex vivo and in vivo findings and our previous report (17), we employed an ILC2-induced AHR model with anti-ICOS antibody and observed that mice treated with anti-ICOS antibody exhibited significantly improved AHR compared with controls (Supplemental Figure 4, A–K). Together, these results suggest that ICOS controls IL-10 production by regulating MAF and NFIL3 expression.

### ICOS regulates IL-10 production via cholesterol biosynthesis.

**Figure 4:**
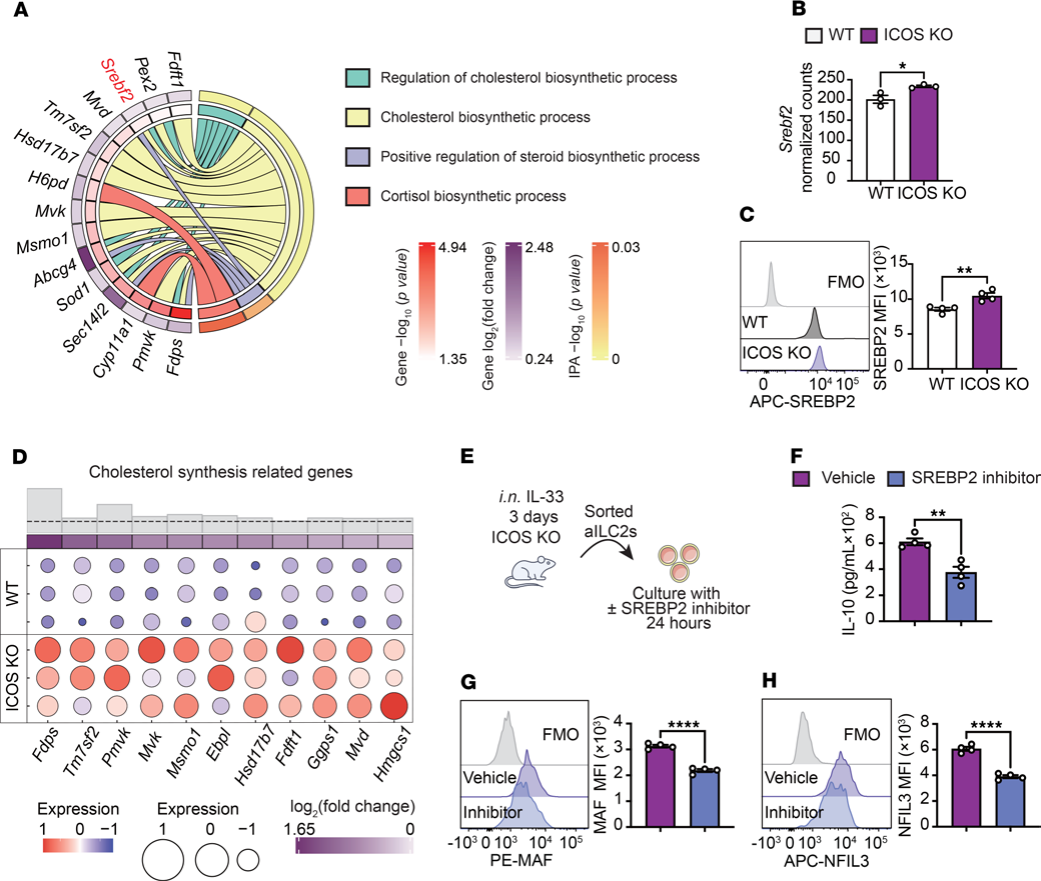
ICOS is related with cholesterol biosynthesis. (**A**) Chord plot representing the differentially expressed genes from the most enriched metabolic pathways. Specific pathways are color-coded and represented in the right inner bands, where chords gather. Outer bands on the right depict the IPA −log_10_ (*P* value). The left inner bands represent the gene −log_10_ (*P* value). Outer bands on the left represent the gene log_2_(fold change). (**B**) The bar graphs represent the normalized counts of *Srebf2*. *n* = 3. (**C**) Representative histogram of protein expression of SREBP2**.** Corresponding quantitation is presented as MFI. *n* = 4. (**D**) Dot plot representing selected critical genes involved in cholesterol synthesis. Dot size is indicative of the total gene expression level. Gray histograms represent −log_10_ (*P* value), and the dotted line represents *P* < 0.05. (**E**–**H**) ICOS-KO aILC2s were cultured with vehicle or SREBP2 inhibitor. (**F**) Levels of IL-10 in the culture supernatant were measured. *n* = 4. (**G** and **H**) Representative plots of MAF (**G**) and NFIL3 (**H**) expression levels in each group are shown. Corresponding quantitation is presented as MFI. *n* = 4. Data are presented as mean ± SEM and are representative of 4 independent experiments. Two-tailed Student’s *t* test or 1-way ANOVA followed by Tukey’s post hoc tests was employed for statistical analysis; *P < 0.05, **P < 0.01, ***P < 0.001, and ****P < 0.0001. Schematic images were created in Adobe Illustrator. FMO, fluorescence minus one.

Recent reports have indicated that the secretion of cytokines by ILC2s is subject to intracellular metabolic reprogramming (7, 9, 28). In our transcriptomic analysis, an Ingenuity Pathway Analysis (IPA) revealed alterations in gene sets associated with cholesterol biosynthetic process, steroid hormone biosynthesis, and cortisol biosynthesis (Figure 4A). We found that the RNA expression of *Srebf2*, a crucial gene in cholesterol biosynthesis (29), is significantly upregulated in ICOS-KO ILC2s (Figure 4B). In confirmation of these observations, we found that the protein expression of SREBP2, which is encoded *Srebf2*, was significantly increased in ICOS-KO ILC2s compared with WT ILC2s (Figure 4C). In addition, the expression levels of a specific set of genes related to cholesterol biosynthesis, which are located downstream of the cholesterol biosynthesis process, were found to be significantly elevated in ICOS-KO ILC2s in comparison with their WT counterparts (Figure 4D). To further investigate the hypothesis that SREBP2 regulates IL-10 production in ICOS-deficient ILC2s, we treated purified populations of activated lung ICOS-KO ILC2s with either vehicle or Fatostatin (30, 31), an SREBP2 inhibitor, for 24 hours (Figure 4E). Treatment with the SREBP2 inhibitor resulted in a significant reduction in IL-10 production compared with the vehicle group (Figure 4F). Similarly, expression of the key transcription factors MAF (Figure 4G) and NFIL3 (Figure 4H) was also decreased following SREBP2 inhibition. Importantly, annexin V staining confirmed that the SREBP2 inhibitor treatment did not induce toxicity (Supplemental Figure 5A). These findings suggest that ICOS may influence cholesterol biosynthesis and IL-10 production in ILC2s by modulating SREBP2 activity.

**Figure 5:**
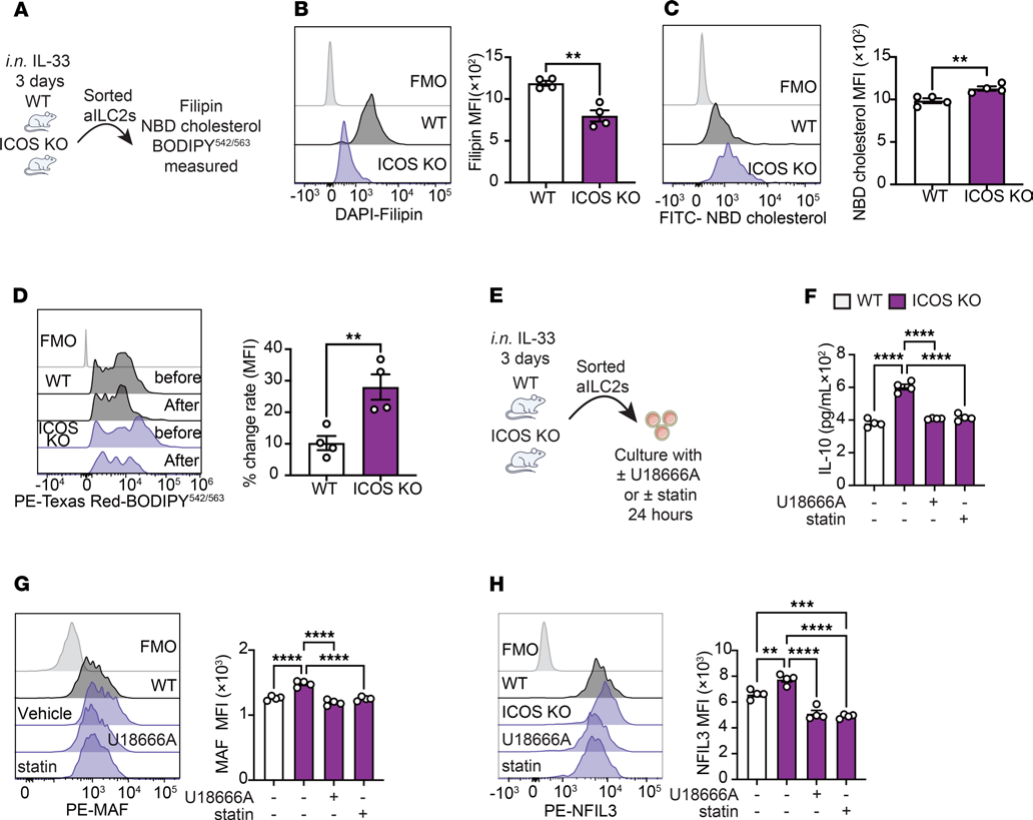
ICOS controls cholesterol biosynthesis in ILC2s for IL-10 production. (**A**) Pulmonary aILC2s were isolated from WT and ICOS-KO mice subjected to i.n. IL-33 challenge for 3 days and cultured. Subsequently, aILC2s were analyzed using Filipin, NBD cholesterol, or BODIPY^542/563^. (**B**) Representative histogram of cholesterol quantity assessed with Filipin fluorescent tracer. Corresponding quantitation is presented as MFI. *n* = 4. (**C**) Representative histogram of NBD cholesterol uptake assessed with a cholesterol uptake kit. Corresponding quantitation is presented as MFI. *n* = 4. (**D**) Representative histogram of lipid utilization assessed with BODIPY^542/563^. Corresponding percent change in MFI before and after incubation is presented. *n* = 4. (**E**–**H**) WT and ICOS-KO aILC2s were cultured with or without U18666A or statin. (**F**) Levels of IL-10 in the culture supernatant were measured. *n* = 4. (**G** and **H**) Representative plots of MAF (**G**) and NFIL3 (**H**) expression levels in each group are shown. Corresponding quantitation is presented as MFI. *n* = 4. Data are presented as mean ± SEM and are representative of 4 independent experiments. Two-tailed Student’s *t* test or 1-way ANOVA followed by Tukey’s post hoc tests was employed for statistical analysis; *P < 0.05, **P < 0.01, ***P < 0.001, and ****P < 0.0001. Schematic images were created in Adobe Illustrator. FMO, fluorescence minus one.

Next, we conducted a comparative analysis of intracellular cholesterol levels to measure the impact of ICOS on cholesterol biosynthesis and usage at the protein level. Activated lung ILC2s were isolated from WT and ICOS-KO mice and cultured for 24 hours. Subsequently, we used Filipin probe to measure the level of intracellular cholesterol, 22-(N-(7-Nitrobenz-2-Oxa-1,3-Diazol-4-yl)Amino)-23,24-Bisnor-5-Cholen-3β-Ol (NBD) cholesterol to measure cholesterol uptake, and BODIPY^542/563^ probes to measure cholesterol usage by flow cytometry (Figure 5A). We observed that the amount of intracellular cholesterol in ICOS-KO ILC2s was diminished in comparison with WT ILC2s, indicating enhanced cholesterol utilization in ICOS-KO ILC2s (Figure 5B). Subsequently, both cholesterol uptake (Figure 5C) and usage (Figure 5D) were upregulated in ICOS-KO ILC2s. These results suggest that ICOS-deficient ILC2s have decreased intracellular cholesterol levels due to increased cholesterol utilization, as indicated by increased NBD-labeled cholesterol levels and changes in BODIPY^542/563^ probes. We next investigated the impact of this higher cholesterol demand in ICOS-KO ILC2s on the production of IL-10 ex vivo using U18666A, a cholesterol transport inhibitor, and statin, a cholesterol synthesis inhibitor. Pure populations of activated lung WT and ICOS-KO ILC2s were cultured with U18666A or statin for 24 hours (Figure 5E). Remarkably, we found that both U18666A and statin reduced IL-10 production in ICOS-KO ILC2s compared with controls (Figure 5F). Similarly, both reagents reduced the expression of MAF (Figure 5G) and NFIL3 (Figure 5H) compared with controls. Of note, the absence of cellular toxicity for U18666A and statin was confirmed by an annexin V assay (Supplemental Figure 5B). Together, these results indicate that the demand for cholesterol is elevated in ICOS-KO and that suppressing cholesterol usage and biosynthesis modulates MAF and NFIL3 expression and ultimately decreases IL-10 production.

### Cortisol biosynthesis induces IL-10 production in ICOS-deficient ILC2s.

**Figure 6:**
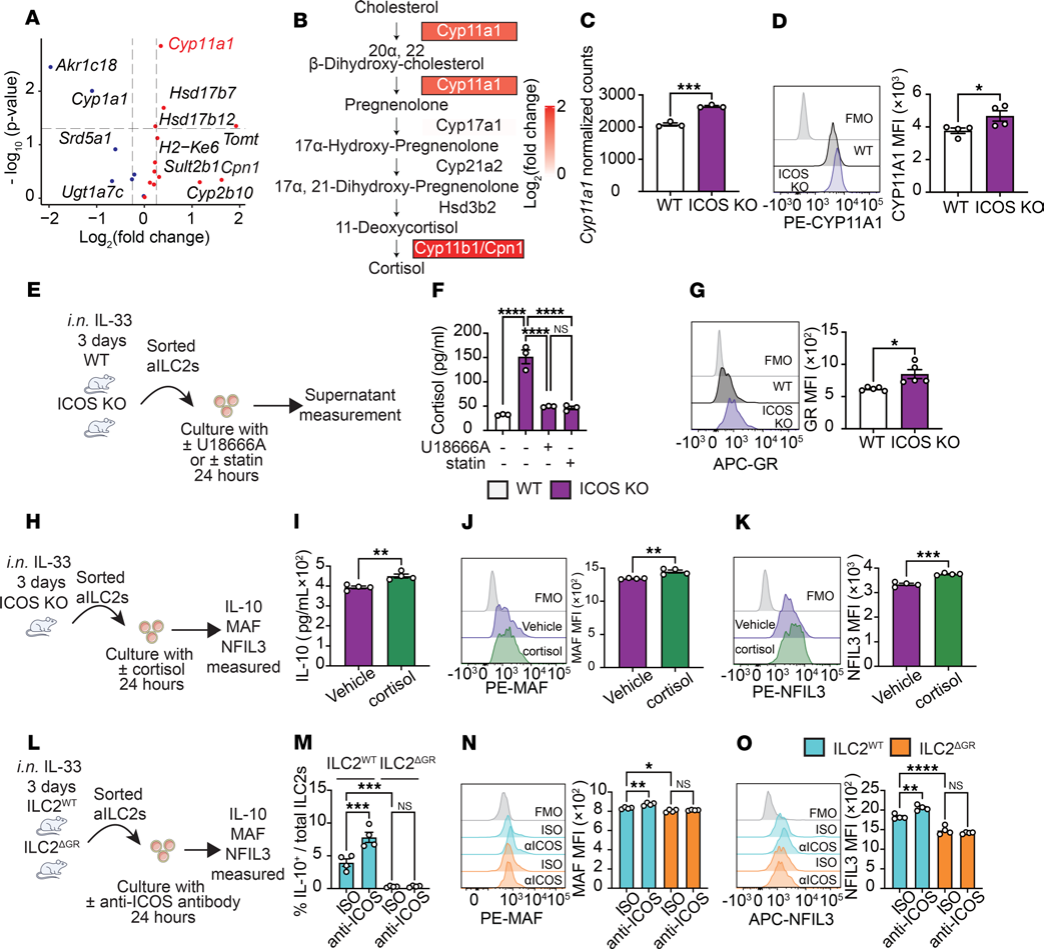
Cortisol is a key regulator of ICOS-mediated IL-10 production in ILC2s. (**A** and **B**) Total RNA from WT and ICOS-KO mice was extracted to perform a bulk transcriptomic analysis. (**A**) Volcano plots represent differentially expressed genes involved in steroid biosynthesis. (**B**) Biosynthetic pathway of cortisol from cholesterol showing intermediate metabolites and key enzymes involved in each step. Changes in enzyme expression are indicated as log_2_(fold change) values in colored boxes. (**C**) The bar graph represents the normalized counts of *Cyp11a1*. *n* = 3. (**D**) Representative histogram of protein expression of CYP11A1. Corresponding quantitation is presented as MFI. *n* = 4. (**E**) WT and ICOS-KO aILC2s were isolated and cultured with or without U18666A or statin. (**F**) Levels of cortisol in the culture supernatant were measured by ELISA. *n* = 3. (**G**) Representative histogram of protein expression of GR. Corresponding quantitation is presented as MFI. *n* = 4. (**H**–**K**) ICOS-KO aILC2s were cultured with or without cortisol. (**I**) Levels of IL-10 in the culture supernatant were measured. *n* = 4. (**J** and **K**) Representative plots of MAF (**J**) and NFIL3 (**K**) expression levels in each group are shown. Corresponding quantitation is presented as MFI. *n* = 4. (**L**–**O**) Sorted aILC2s from NMUR1^cre^ (ILC2^WT^ mice) and NMUR1^cre^GR^fl/fl^ mice (ILC2^ΔGR^ mice) were cultured with isotype control or anti-ICOS antibody. (**M**) Bar graph representing the frequency of IL-10^+^ ILC2s in each group. *n* = 4. (**N** and **O**) Representative plots of MAF (**N**) and NFIL3 (**O**) expression levels in each group are shown. Corresponding quantitation is presented as MFI. *n* = 4. Data are presented as mean ± SEM and are representative of 4 independent experiments. Two-tailed Student’s *t* test was employed for statistical analysis; *P < 0.05, **P < 0.01, and ***P < 0.001, and ****P < 0.0001. Schematic images were created in Adobe Illustrator. FMO, fluorescence minus one.

Cholesterol serves as the essential precursor molecule for the biosynthesis of cortisol through a series of enzymatic reactions (32). In particular, the analysis of the transcriptome revealed that, among the genes related to steroid hormone biosynthesis, the *Cyp11a1* gene, which plays an important role in cortisol biosynthesis (33), is increased in ILC2 lacking ICOS (Figure 6A). In addition, the results of the IPA depicted in Figure 6A demonstrated that the gene set involved in cortisol biosynthetic process was overexpressed in ICOS-KO ILC2s. Consequently, we examined the gene expression of enzymes associated with cholesterol biosynthesis and observed that the genes detected were also overexpressed (Figure 6B). This finding was corroborated by the elevated *Cyp11a1* expression levels in ICOS-KO ILC2s at both the RNA and protein levels when compared with WT ILC2s (Figure 6, C and D). Furthermore, the expression of CYP11A1 was significantly upregulated in ICOS-KO ILC2s compared with WT ILC2s in the *Alternaria* stimulation model (Supplemental Figure 5, C and D). Based on these findings, we hypothesized that the biosynthesis of cholesterol is enhanced in ICOS-KO ILC2s and that cholesterol is used to biosynthesize cortisol. To investigate this hypothesis, we conducted an experiment to examine whether ICOS-KO or WT ILC2s produce cortisol using cholesterol inhibitors or cholesterol transport inhibitors. Pure populations of activated WT and ICOS-KO ILC2s were treated with or without U18666A or statin, and the cortisol level was measured in the culture supernatant by ELISA (Figure 6E). Strikingly, ICOS-KO ILC2s produced significantly more cortisol compared with controls, whereas U18666A and statin led to a decrease in cortisol production (Figure 6F). Subsequently, to ascertain the impact of cortisol on ILC2s, we examined the expression of GR, a receptor for cortisol, in ILC2s. This investigation revealed that GR is expressed in ILC2s, with significantly higher expression in ICOS-KO ILC2s compared with WT ILC2s (Figure 6G). Similar results were observed in the *Alternaria* stimulation model (Supplemental Figure 5, C and E). We next investigated the effects of cortisol on IL-10 production in ILC2s and exposed activated ICOS-KO ILC2s with cortisol ex vivo for 24 hours (Figure 6H). We found that cortisol induced IL-10 production in ILC2s (Figure 6I), accompanied by increased intranuclear MAF and NFIL3 expressions (Figure 6, J and K). To further validate this observation, ILC2^ΔGR^ mice, which lack GR specifically in ILC2s, were generated by crossing Nmur1Cre^+/–^ mice (ILC2^WT^ mice) and GR^fl/fl^ mice. We confirmed NMUR1 is specifically expressed in ILC2s in the lungs and not expressed on other pulmonary immune cells such as T cells or eosinophils in our context (Supplemental Figure 6A). To investigate the role of GR in ILC2 development and homeostasis, we quantified naive ILC and ILC2 populations under steady-state conditions and following IL-33–induced activation using ILC2^WT^ and ILC2^ΔGR^ mice. At steady state, no significant differences were observed in total ILC numbers (lineage^–^IL-7R^+^ cells), IL-7R expression on ILCs, or ILC2 frequencies between the 2 groups. In contrast, IL-33 stimulation resulted in a significantly greater expansion of ILC2s in ILC2^ΔGR^ mice compared with ILC2^WT^ mice, accompanied by increased ILC2 number and elevated IL-7R expression. These findings indicate that GR deficiency does not impair ILC2 development but enhances ILC2 activation in response to IL-33 (Supplemental Figure 6, B–D). Activated ILC2s were sorted from the lungs of ILC2^WT^ and ILC2^ΔGR^ mice and cultured with an anti-ICOS antibody and isotype control (Figure 6L). As expected, the frequency of IL-10 producing ILC2s was increased in WT ILC2s incubated with anti-ICOS. Remarkably however, IL-10 production drastically decreased in ILC2s isolated from ILC2^ΔGR^ mice compared with controls, while no effects of anti-ICOS on IL-10 production were observed in these mice (Figure 6M). Correspondingly, MAF and NFIL3 intranuclear expressions also showed similar changes (Figure 6, N and O). Consistent with previous findings, GR-deficient ILC2s in the *Alternaria* stimulation model exhibited a similar downregulation of MAF and NFIL3 expression (Supplemental Figure 6, E–G). These observations were further confirmed using Mifepristone, a GR inhibitor (34) (Supplemental Figure 7, A–D). Together, these results suggest that cortisol promotes IL-10 production in ILC2s via GR expression located downstream of ICOS.

### ILC2 conditional deletion of GR exacerbates the development of ILC2-dependent AHR.

**Figure 7:**
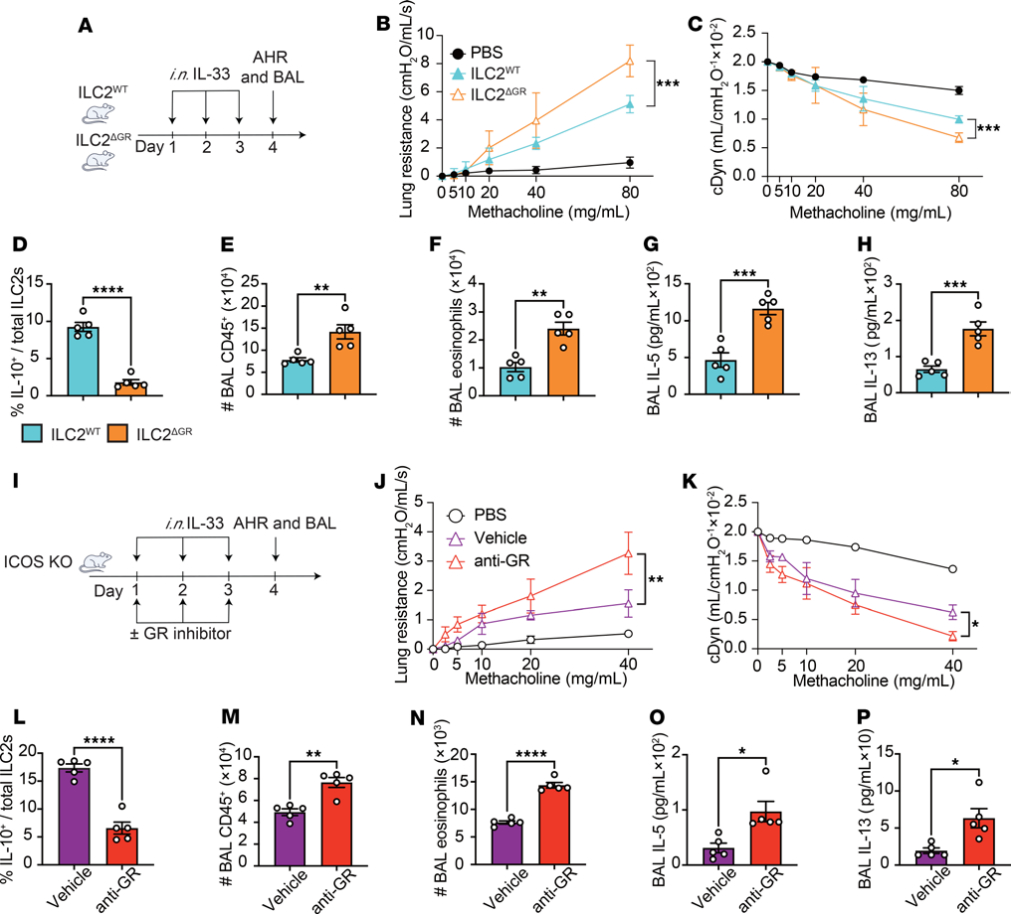
ILC2-specific GR deletion or inhibition in ICOS-KO mice exacerbates the ILC2-induced AHR. (**A**–**H**) NMUR1^cre^ (ILC2^WT^ mice) and NMUR1^cre^GR^fl/fl^ mice (ILC2^ΔGR^ mice) were i.n. exposed to 0.5 μg of rmIL-33 or PBS for 3 days. On day 4, lung function, frequency of IL-10^+^ ILC2s in the lung, BAL cellularity, cytokine levels, and lung histology were analyzed. (**B** and **C**) Lung resistance (**B**) and dynamic compliance (**C**) in response to elevating doses of methacholine. *n* = 5. (**D**–**F**) The frequency of IL-10^+^ ILC2s in the lung (**D**), the total number of CD45^+^ cells **(E),** and CD45^+^, Gr-1^–^, CD11c^–^, and SiglecF^+^ eosinophils (**F**) in BAL fluid are demonstrated in bar graphs. *n* = 5. (**G** and **H**) Levels of IL-5 (**G**) and IL-13 (**H**) in the BAL fluid are shown in bar graphs. *n* = 5. (**I**–**P**) ICOS-KO mice were i.n. exposed to 0.5 μg of rmIL-33 or PBS with or without GR inhibitor (0.01nM) (anti-GR) for 3 days. On day 4, lung function, frequency of IL-10^+^ ILC2s in the lung, BAL cellularity, cytokine levels, and histology were analyzed. (**J** and **K**) Lung resistance (**J**) and dynamic compliance (**K**) in response to elevating doses of methacholine. *n* = 5. (**L**–**N**) The frequency of IL-10^+^ ILC2s in the lung (**L**), total number of CD45^+^ cells (**M**), and CD45^+^, Gr-1^–^, CD11c^–^, and SiglecF^+^ eosinophils (**N**) in BAL fluid are demonstrated in bar graphs. *n* = 5. (**O** and **P**) Levels of IL-5 (**O**) and IL-13 (**P**) in the BAL fluid are shown in bar graphs. *n* = 5. Data are presented as mean ± SD or SEM and are representative of 3 independent experiments. Two-tailed Student’s *t* test or 1-way ANOVA followed by Tukey’s post hoc tests was employed for statistical analysis; *P < 0.05, **P < 0.01, ***P < 0.001, and ****P < 0.0001. Schematic images were created in Adobe Illustrator.

We next sought to investigate the role of GR and IL-10 production in the development of ILC2-driven AHR. ILC2^WT^ and ILC2^ΔGR^ mice were administered i.n. with IL-33 or PBS for 3 consecutive days. On the fourth day, lung resistance and dynamic compliance were measured by noninvasive plethysmography, followed by the analysis of BAL cellularity by flow cytometry and lung histology (Figure 7A). We found that ILC2^ΔGR^ mice exhibited significantly higher pulmonary resistance compared with ILC2^WT^ mice (Figure 7B), associated with a decrease dynamic compliance (Figure 7C). In line with our previous findings, the percentage of IL-10^+^ ILC2s isolated from the lungs of ILC2^ΔGR^ mice was significantly decreased compared with ILC2^WT^ mice (Figure 7D). The frequency of IL-5^+^ IL-13^+^ ILC2 was then assessed, and no statistically significant differences were found between the 2 groups (Supplemental Figure 8A). This observation was associated with increased inflammation, as indicated by the higher numbers of CD45^+^ cells, notably eosinophils (Figure 7, E and F), and the higher levels of IL-5 and IL-13 (Figure 7, G and H) in the BAL fluid of ILC2^ΔGR^ mice. Histological analysis of lung tissue (Supplemental Figure 8B) corroborated these results, demonstrating that IL-33 challenges led to a substantial increase in epithelial thickness (Supplemental Figure 8C) and inflammatory cell count (Supplemental Figure 8D) in ILC2^ΔGR^ mice compared with ILC2^WT^ mice. Together, these findings support the notion that GR in ILC2s controls the magnitude of AHR, independently of other immune cells.

### GR depletion in ICOS-KO mice exacerbates ILC2-dependent AHR.

To find a possible link between ICOS and GR effects on ILC2s, we conducted experiments in ICOS-KO mice using GR inhibitor (34). ICOS-KO mice were challenged i.n. with IL-33 or PBS, in the presence or absence of GR inhibitor (0.01 nM) (anti-GR) on days 1–3 (Figure 7I), and on day 4, lung resistance and dynamic compliance were directly measured, followed by the analysis of BAL cellularity by flow cytometry and lung histology. We found that lung resistance was significantly higher in mice treated with GR inhibitor compared with controls (Figure 7J), associated with the worst dynamic compliance (Figure 7K). In confirmation of our previous findings, blocking GR in ICOS-KO mice reduced the frequency of IL-10–producing ILC2s in the lungs (Figure 7L), associated with an increase in inflammation, as evidenced by the number of CD45^+^ cells (Figure 7M) and eosinophils (Figure 7N) as well as IL-5 (Figure 7O) and IL-13 (Figure 7P) levels in the BAL fluid. Histological analysis of lung tissue (Supplemental Figure 8E) corroborated these results, demonstrating that epithelial thickness (Supplemental Figure 8F) and inflammatory cell count (Supplemental Figure 8G) were higher in mice treated with GR inhibitor compared with vehicle mice. These results suggest that the inhibition of GR in ICOS-KO may promote AHR exacerbation in the presence of IL-33, a conclusion further supported by experiments using *A*. *alternata*, a common fungus associated with allergic disease (Supplemental Figure 9, A–K). These findings suggest that ICOS exerts a regulatory effect on IL-10 production by suppressing the activity of GR in ILC2, thereby modulating AHR.

### The ICOS/GR pathway regulates IL-10 production and effector function in hILC2s.

**Figure 8:**
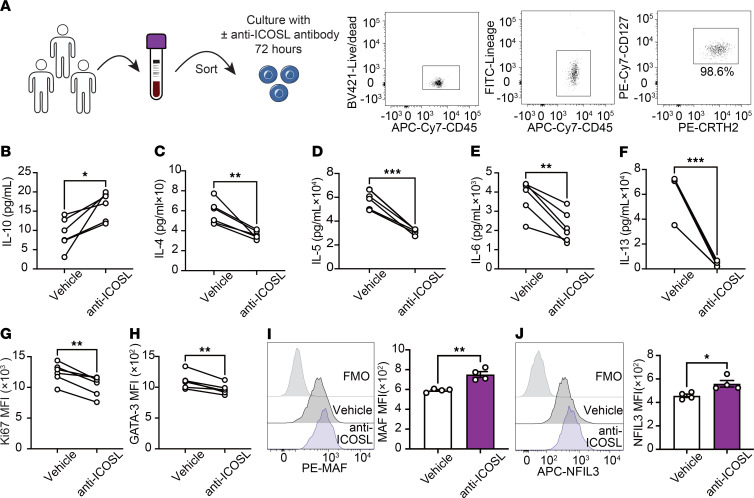
ICOS regulates IL-10 production in hILC2s. (**A**–**H**) hILC2s (CD45^+^, lineage^–^, CRTH2^+^, and CD127^+^) were freshly isolated from PBMCs of healthy donors and cultured with or without anti-ICOS ligand (anti-ICOSL). Right panel shows hILC2 purity after being sorted. (**B**–**F**) Levels of IL-10 (**B**), IL-4 (**C**), IL-5 (**D**), IL-6 (**E**), and IL-13 (**F**) in the culture supernatants following treatment with or without anti-ICOSL. *n* = 6. (**G** and **H**) The expression levels of intranuclear Ki67 (**G**) and GATA-3 (**H**) expression is presented as MFI. *n* = 6. (**I** and **J**) Representative histogram of MAF (**I**) and NFIL3 (**J**) protein expression levels**.** Corresponding quantitation is presented as MFI. *n* = 4. Data are presented as mean ± SEM. Two-tailed Student’s *t* test was employed for statistical analysis; *P < 0.05, **P < 0.01, and ***P < 0.001. Schematic images were created in Adobe Illustrator. FMO, fluorescence minus one.

**Figure 9:**
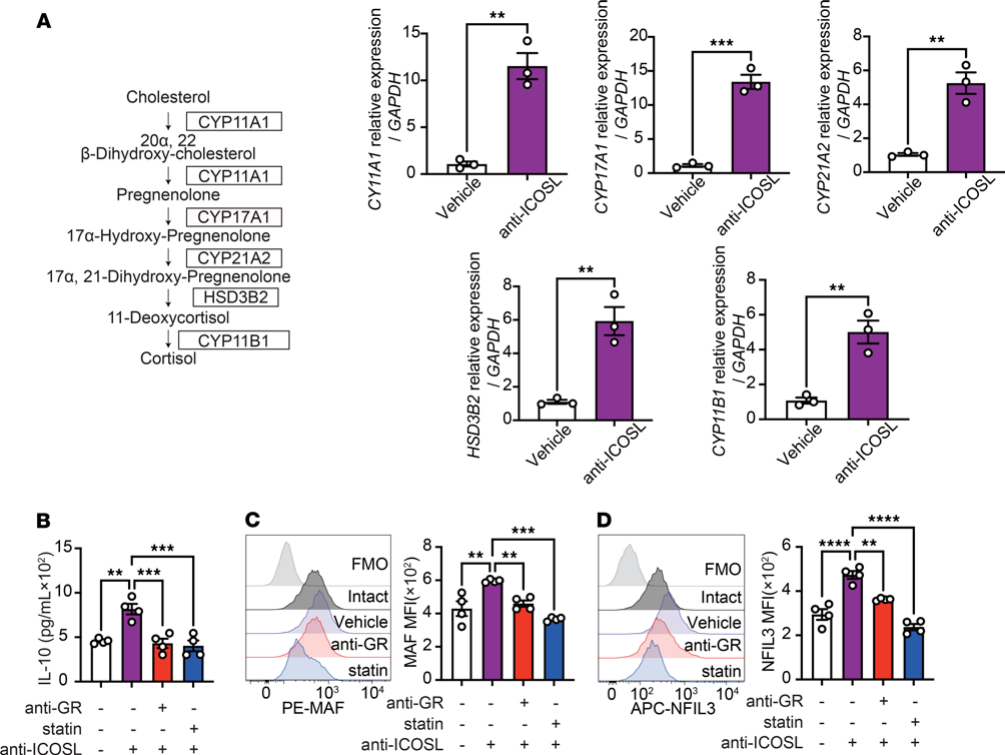
ICOS regulates IL-10 production in hILC2s via controlling cholesterol and cortisol biosynthesis. (**A**) qRT-PCR results show *CYP11A1*, *CYP17A1*, *CYP21A2*, *HSD3B2*, and *CYP11B1* expression in each group. *n* = 3. (**B**) Levels of IL-10 in the culture supernatants following treatment with or without GR inhibitor (anti-GR) or statin. *n* = 4. (**C** and **D**) Representative histogram plots of intranuclear MAF (**C**) and NFIL3 (**D**) expression levels and corresponding quantitation presented as MFI. *n* = 4. Data presented as mean ± SEM. Two-tailed Student’s *t* test or 1-way ANOVA followed by Tukey’s post hoc tests was employed for statistical analysis; *P < 0.05, **P < 0.01, ***P < 0.001, and ****P < 0.0001. Schematic images were created in by Adobe Illustrator. FMO, fluorescence minus one.

We then sought to determine whether the observations from the rodent studies were applicable to hILC2s. We isolated pure populations of hILC2s from PBMCs of 6 healthy individuals as CD45^+^, lineage^–^, CD127^+^, and CRTH2^+^ cells using flow cytometry (Figure 8A and Supplemental Figure 10). We previously reported that ICOS and ICOSL are expressed in both murine and hILC2s (17). We then cultured hILC2s isolated from each healthy subject with or without anti-human ICOSL antibody (anti-ICOSL) for blocking the ICOS–ICOSL interaction and examined the effects of ICOSL blockade on ILC2 activation, proliferation, and functional indices (Figure 8A). The administration of the anti-ICOSL led to a significant increase in IL-10 levels in the culture supernatant of all 6 healthy subjects. In addition, consistent with prior reports, hILC2s treated with anti-ICOSL showed a decrease in the levels of effector cytokines, including IL-4, IL-5, IL-6, and IL-13, as well as the intranuclear proteins Ki67, a measure of hILC2 proliferation, and GATA-3, a hallmark activation marker for hILC2s (Figure 8, B–H). We next sought to elucidate the role of ICOS, cholesterol, and cortisol on IL-10 production using anti-ICOSL, statin, or GR inhibitor (anti-GR) in hILC2 cultures. The expression of MAF (Figure 8I) and NFIL3 (Figure 8J) in hILC2 was increased by anti-ICOSL treatment, aligning with our observations from murine ILC2s. Furthermore, the genes associated with cortisol biosynthesis–related enzymes, as illustrated in Figure 6B, included the following: CYP11A, which catalyzes the conversion of cholesterol to pregnenolone; CYP17A1, which converts pregnenolone to 17α-hydroxy-pregnenolone; CYP21A2, which converts 17α-hydroxy-pregnenolone to 17α,21-dihydroxy-pregnenolone; HSD3B2, which converts 17α,21-dihydroxy-pregnenolone to 11-deoxycortisol; and CYP11B, which facilitates the transformation of 11-deoxycortisol to cortisol (Figure 9A). These enzymes were found to be significantly upregulated by anti-ICOSL treatment (Figure 9A). In addition, and consistent with our observations in murine models, the administration of GR inhibitor or statins led to a substantial decrease in IL-10 production (Figure 9B) and MAF (Figure 9C) and NFIL3 (Figure 9D) expression.

Taken together, these results align with those previously observed in murine models, suggesting a conserved mechanism through which ICOS regulates IL-10 production in hILC2s by modulating cholesterol and cortisol biosynthesis.

## Discussion

The central finding of this study is that in pulmonary ILC2s, ICOS negatively controls the production of the anti-inflammatory cytokine IL-10 by regulating cholesterol and cortisol biosynthesis. We found that the anti-inflammatory cytokine IL-10 was upregulated in the absence of ICOS in ILC2s ex vivo, as we used an anti–IL-10R antibody to specifically demonstrate the immunomodulatory role of IL-10 on the function of ILC2s and development of AHR in multiple mouse models. Remarkably, in the absence of ICOS signaling, ILC2s accumulated increased amounts of cholesterol ex vivo, as measured by Filipin and NBD cholesterol assays. A combination of transcriptomic and protein analysis further showed that ILC2s lacking ICOS increased CYP11A1, an enzyme involved in the production of cortisol from cholesterol, which led to higher amounts of intracellular cortisol in ILC2s and an increase in cortisol receptor GR expression, an effect neutralized by statins. We further found that MAF and NFIL3, 2 transcription factors known to positively control IL-10 production, were similarly upregulated in the absence of ICOS in ILC2s. To confirm our findings in an in vivo setting, we generated mice with an ILC2-specific deletion of GR. We found that ILC2s lacking GR were unable to induce IL-10 upon ICOS inhibition, associated with the failure to upregulate MAF and NFIL3. In support of these findings, mice lacking ICOS and treated with an anti-GR antibody showed similarly increased development of AHR compared with controls, which was associated with lower IL-10 production by ILC2s in the lungs in multiple mouse models. To the best of our knowledge, this is the first report showing that ICOS regulates IL-10 production by controlling steroid metabolism in ILC2s.

ICOS is a costimulatory molecule expressed primarily on immune cells, particularly Tregs (35). ICOS molecules possess intracellular signals that modulate immune cell function, and the induction of ICOS signaling can have distinct effects depending on the immune cells and the context. For instance, in Th2 cells, ICOS enhances allergic responses by promoting the production of Th2 cytokines (15). In contrast, in Tregs, ICOS has been shown to promote the production of IL-10, an anti-inflammatory cytokine, thereby facilitating immune tolerance (14). Notably, recent work by O’Brien et al. further illustrated this context specificity, demonstrating that ICOS can differentially regulate IL-10 production in T cells depending on the immune environment, such as during chronic infection, highlighting its intricate role in modulating immune responses (36). Collectively, these observations underscore the critical and nuanced regulatory functions of ICOS in orchestrating immune allergic responses within the immune system. In our previous report, we demonstrated the presence of ICOS and ICOSL in ILC2s, and the interaction between ICOS and ICOSL led to the production of Th2 cytokines such as IL-5 and IL-13. In this study, we demonstrated that the suppression of ICOS in pulmonary ILC2s resulted in the secretion of IL-10, a cytokine that is recognized for its anti-inflammatory properties. This finding is further corroborated by reports of reduced ICOS expression in T cells and enhanced IL-10 production in chronic inflammation (37, 38).

Recent reports have indicated a regulatory role for cholesterol in the production of IL-10 in immune cells (39, 40). For instance, activated CD4^+^ Th1 cells have been observed to be associated with the expression of a group of cholesterol metabolism genes, exhibiting a shift from IFN-γ production to IL-10 production (39). In addition, cholesterol metabolism in regulatory B cells has been documented as a critical metabolic pathway for optimal function of IL-10–producing regulatory B cells (40). In this study, we demonstrate that IL-10 production is enhanced in ICOS-KO ILC2s compared with WT ILC2s. Our investigations notably revealed augmented cholesterol biosynthesis and utilization in ILC2s lacking ICOS compared with controls. In support of our findings, we observed that IL-10 production was suppressed when ICOS-KO ILC2s were treated with inhibitors of cholesterol biosynthesis (statin) and cholesterol utilization (U18666A). These findings support those previously reported for Th1 cells and support the involvement of cholesterol metabolism in the IL-10 production mechanism in ILC2s (39).

Cortisol, a well-known hormone, is synthesized from cholesterol through a series of metabolic processes involving multiple enzymes (32, 41). Cortisol is thought to be involved in the production of IL-10 in various immune cells, but reports differ depending on the immune cell and context. For instance, it has been documented that cortisol can induce the release of IL-10 from PBMCs (42). Conversely, in B cells, patients with atherosclerosis and elevated cortisol levels exhibit a reduced percentage of IL-10–producing B cells, suggesting that cortisol may inhibit IL-10 production in this cell type (43). Furthermore, an indirect role of cortisol in T cells has been reported where cortisol acts on memory CD4^+^ T cells to promote IL-10 production (44). Our findings show that cortisol secreted by ILC2s can promote IL-10 production in ILC2s. These effects are driven by the lack of ICOS signaling, supporting the notion that ICOS may directly repress cortisol biosynthesis in ILC2s. These findings are consistent with previous studies on antigen-presenting cells and CD4^+^ T cells, which demonstrated that cortisol stimulates IL-10 production (44). Furthermore, ILC2s were shown to secrete small amounts of cortisol, suggesting that it may contribute to lung homeostasis by affecting other ILC2s and antigen-presenting cells in negligible amounts, as well as amphiregulin, previously secreted in negligible amounts from ILC2s (45, 46).

Several transcription factors, including MAF and NFIL3, have been identified as regulators of IL-10 expression (47). Specifically, MAF has been documented to stimulate IL-10 production in a range of immune cell types, including Th17 and B cells, as well as ILC2s (12, 48, 49). NFIL3 has also been reported as a protein that regulates IL-10 production in Th1 and Th2 cells (50, 51). Interestingly, MAF and NFIL3 are both glucocorticoid-regulated transcription factors, as both MAF (52) and NFIL3 (51) were described to be regulated by GR. In this study, we combined a comprehensive transcriptome analysis of ICOS-KO versus WT lung ILC2s and ILC2-specific GR-KO mice to clarify the role of MAF, NFIL3, and GR in ILC2-driven IL-10 production. Additionally, the administration of anti-ICOS antibody to ILC2-specific GR-KO ILC2s did not alter their ability to produce IL-10 or affect the expression of MAF and NFIL3. Consequently, our findings indicate that GR is downstream of ICOS signaling and upstream of MAF and NFIL3. While our data in Supplemental Figure 6A indicate that GR signaling in ILC2s does not directly suppress their intrinsic IL-5/IL-13 production, we observed increased levels of IL-5 and IL-13 in the BAL fluid of GR-KO ILC2 mice (Figure 7, G and H) alongside reduced IL-10 production by ILC2s. This suggests that the exacerbated AHR in these mice may not solely be due to changes in ILC2-derived type 2 cytokines. It is plausible that the reduced IL-10 from ILC2s, which we demonstrate plays a critical immunomodulatory role, allows for an unconstrained contribution of IL-5 and IL-13 from other immune cell types, such as T cells, which are known to be significant producers of these cytokines. Further studies profiling intracellular cytokine production across various immune cell subsets in this model would be beneficial to fully delineate the cellular sources of these increased type 2 cytokines and their relative contribution to AHR. Therefore, while our data strongly support IL-10 as a key mediator, the interplay with other immune cell contributions to the overall type 2 cytokine milieu and subsequent AHR warrants further investigation and represents a limitation of the current scope. In recent years, there has been a notable rise in the number of patients diagnosed with steroid-resistant asthma (53), highlighting a pressing need for alternative therapeutic strategies. Emerging evidence suggests that ICOS is expressed in steroid-resistant ILC2s (54), pointing to its potential role in mediating resistance. Our findings further indicate a link between ICOS deficiency and increased GR expression, suggesting that ICOS may negatively regulate GR levels and thus contribute to steroid resistance. These observations raise the possibility that targeting ICOS signaling in ILC2s could enhance GR expression and restore steroid sensitivity, offering a promising avenue for the development of novel treatments for steroid-resistant asthma.

In a translational approach, we were able to reduce ILC2 activation and effector function by administering anti-ICOSL antibody to ILC2s isolated from PBMCs of healthy volunteers. Our results support our murine findings and show that the inhibition of ICOS signaling in hILC2s decreases the production of IL-4, IL-5, IL-6, and IL-13, while remarkably increasing that of IL-10. Cortisol biosynthesis was similarly affected by ICOS, as we observed the increased expression of multiple enzymes related to cortisol biosynthesis in hILC2s incubated with anti-ICOSL antibody. Notably, the inhibition of ICOS was found to promote IL-10 production, while the suppression of cholesterol biosynthesis or utilization, and the inhibition of GR, all led to a subsequent inhibition of IL-10 production. Consistent with the findings observed in the murine model, this alteration in IL-10 production exhibited a persistent correlation with MAF and NFIL3 expression. Our results therefore suggest that ICOS regulates IL-10 production by modulating the activation of GR, MAF, and NFIL3 through the biosynthesis of cholesterol and cortisol. Our findings open the door for the design of novel therapies, as targeting ICOS specifically in ILC2s would not only suppress the secretion of inflammatory cytokines, but also promote the production of IL-10, an anti-inflammatory cytokine. Such a therapeutic approach holds promise for the treatment of allergic asthma and airway type 2 inflammation.

Taken together, our findings suggest that ICOS negatively regulates IL-10 production in ILC2s through a metabolic mechanism involving cholesterol and cortisol biosynthesis, with GR activation serving as a key transcriptional regulator. Given the role of ILC2s in allergic airway inflammation, targeting ICOS selectively in these cells could offer a novel therapeutic approach to suppress type 2 inflammation while enhancing IL-10–mediated immune regulation. However, species-specific differences in ILC2 metabolism and the potential systemic effects of ICOS inhibition warrant further investigation. Future studies should explore the precise molecular interactions between ICOS signaling, metabolic pathways, and transcriptional regulators to refine therapeutic strategies targeting ICOS in allergic and inflammatory diseases.

## Methods

### Sex as a biological variable.

Our study examined male and female animals, with similar findings reported for both sexes.

### Animals.

WT BALB/cByJ or C57BL/6J mice, Nmur1Cre^+/–^ mice [C57BL/6-Tg(Nmur1-iCre, -eGFP)1Dart/J], GR^fl/fl^ mice (B6.129S6-Nr3c1tm2.1Ljm/J), and IL-10 GFP mice [B6(Cg)-Il10tm1.1Karp/J], aged 6–8 weeks, were procured from The Jackson Laboratory. ICOS-deficient mice (C.129S4-Icostm1Shr/J) were obtained from Arlene Sharpe (Harvard Medical School, Boston, Massachusetts, USA). Six- to eight-week-old age- and sex-matched mice were used in the studies. Nmur1^cre^GR^fl/fl^ (ILC2^ΔGR^) mice were generated by breeding Nmur1^cre^ (ILC2^WT^) and GR^fl/fl^ mice together. ILC2^ΔGR^ and ILC2^WT^ mice were used in experiments. All mice were housed in a pathogen-free animal facility at the Keck School of Medicine, University of Southern California (USC), in accordance with protocols approved by the IACUC.

### Isolation and ex vivo culture of ILC2s from mouse lungs.

To isolate murine pulmonary ILC2s, mice were i.n. challenged with rmIL-33 (BioLegend; 0.5 μg in 40 μL PBS) for 3 days under anesthesia. On the fourth day, pulmonary ILC2s were sorted using FACS to achieve a purity exceeding 95% on a FACSAria III system (BD Biosciences), as previously described (12). Following transcranial perfusion with PBS to remove circulating cells, the lungs underwent enzymatic digestion using collagenase type IV (400 U/mL; Worthington) at 37°C for 1 hour. The resulting suspensions were processed into a single-cell suspension through a 70 μm cell strainer (Falcon). RBCs were lysed using RBC lysis buffer (BioLegend) before staining. Live single cells that expressed CD45, ST2, and CD127 and did not express lineage markers (CD3ε, CD4, CD5, TCR-β, TCR-γδ, CD45R/B220, CD335, CD11c, CD11b, Gr1, FcεRIα, and Ter119) were considered ILC2s. Isolated ILC2s were cultured ex vivo at 37°C in 96-well U-bottom plates at a density of 510^4^ cell/mL in RPMI medium supplemented with 10% heat-inactivated FBS (Omega Scientific), 100 units/mL penicillin and 100 mg/mL streptomycin (GenClone), rmIL-2 (10 ng/mL; BioLegend), and rmIL-7 (10 ng/mL; BioLegend), henceforth referred to as complete RPMI. For studies involving ICOS blockade, a monoclonal antibody targeting ICOS at a concentration of 10 μg/mL (BE0059; Bio X Cell) was added to cultures for designated durations. For knockdown studies, 2.5 mM Maf 5′-GTTCATTGCCAGTTCTGAAGCCATC-3′, Nfil3 5′-GCTGCATCAGAAGGACCTCCTCGT-3′, or random control oligo (Gene Tools, LLC) was added as free uptake oligo for 24 hours as previously described (12). To inhibit IL-10R signaling, 10 μg/mL of IL-10R antibody (1B1.3A; Bio X Cell) was added to ILC2 cultures for 18–24 hours. To inhibit SREBP2, Fatostatin (125B11; MedChemExpress) (SREBP2 inhibitor, 0.5 μM) was added to ILC2 cultures for either 24 hours or 1 hour. To block cholesterol biosynthesis, Atorvastatin Calcium (NA.24; Sigma-Aldrich Solutions) (statin, 5 μM) and U18666A (NA.77; 2 μg/mL; Sigma-Aldrich Solutions) were added to ILC2 cultures for either 24 hours or 1 hour. To examine the role of hydrocortisone in ILC2s, hydrocortisone was added to the ILC2 culture medium at a concentration of 0.01 nM as a GR stimulator and Mifepristone (NA.77; Sigma-Aldrich Solutions) as a GR inhibitor.

### In vivo experiments on mice and collection of BAL samples.

Mice were i.n. challenged with either 0.5 μg/mouse rmIL-33 or PBS for 3 consecutive days under anesthesia, according to a previous protocol (8). In experiments involving *A*. *alternata*, mice received i.n. doses of 100 μg/mouse *A*. *alternata* for 4 consecutive days under anesthesia, as previously established by our group (10). Twenty-four hours after the final i.n. challenge, lungs tissues were enzymatically digested, processed into single-cell suspensions, and subjected to the designated analyses. For BAL analysis, the airways were ravaged with 1 mL of cold PBS 3 times and aspirated. BAL samples were then centrifuged to collect cells, and the supernatants were preserved for cytokine measurement assays. Prior to flow cytometry analysis, cells harvested from BAL samples underwent treatment with RBC lysis buffer for optimal staining.

### Flow cytometry.

The following panel of murine antibodies were utilized: FITC or APC anti-mouse lineage markers (CD3ε [145-2C11], CD4 [GK1.5], CD5 [53-7.3], TCR-β [H57-597], TCR-γδ [UC7-13D5, GL3], B220/CD45R [RA3-6B2], Gr-1 [RB6-8C5], CD11c [N418], CD11b [M1/70], Ter119 [TER-119], FcεRI [MAR-1], and CD335 [29A1.4]), PE-Cy7 anti-mouse CD127 (A7R34), APC-Cy7 anti-mouse CD45 (30-F11), PE-Cy7 anti-mouse CD45 (30-F11), Brilliant Violet 510 anti-mouse CD45.1 (A20), Alexa Fluor 488 anti-mouse CD45.2 (104), APC-Cy7 anti-mouse CD11c (N418), FITC anti-mouse CD19 (6D5), APC anti-mouse Gr-1 (RB6-8C5), and PerCP-Cy5.5 anti-mouse CD3e (17A2), all acquired from BioLegend. Additionally, PE anti-mouse SiglecF (E50-2440) and PE anti-mouse CD278 (ICOS) (7E.17Gg) were purchased from BD Biosciences, and PerCP-eFluor710 anti-mouse ST2 (RMST2-2) and eFluor450 anti-mouse CD11b (M1/70) were obtained from Thermo Fisher Scientific. When indicated, the Cholesterol Cell-Based Detection Assay Kit (Cayman Chemical), Cholesterol Uptake Assay Kit (Abcam), and BODIPY^542/563^ (Thermo Fisher Scientific) were employed as per the manufacturers’ instructions.

Cells were stimulated for 4 hours ex vivo with 50 ng/mL PMA, 500 ng/mL ionomycin (both from Sigma), and 1 μg/mL Golgi plug (BD Biosciences). PE or eFluor 450 anti-mouse IL-13 (eBio13A; Thermo Fisher Scientific), APC anti-mouse IL-5 (TRFK3; BioLegend), APC anti-mouse IL-10 (JES5-16E3; BioLegend), and PE anti-mouse IL-10 (JES5-16E3; BD Biosciences) were used for intracellular cytokine staining. For intranuclear staining, the Foxp3 Transcription Factor Staining Kit (Thermo Fisher Scientific) was used along with APC anti-mouse Ki67 (SolA15; Thermo Fisher Scientific), PE anti-mouse/human GATA-3 (TWAJ; Thermo Fisher Scientific), APC anti-mouse NR3C1 (BuGR2; Thermo Fisher Scientific), PE anti-human and -mouse cMAF (symOF1; Thermo Fisher Scientific), PE or APC anti-mouse NFIL3 (S2M-E19; Thermo Fisher Scientific), APC anti-human NFIL3 (MABA223; Thermo Fisher Scientific), APC anti-human and -mouse SREBF2 (D9889; Biorbyt), and PE anti-mouse CYP11a1 (BC05787763; Bioss). Apoptosis staining was performed using PE annexin V (Thermo Fisher Scientific) and DAPI (Sigma), based on the manufacturers’ instructions.

The following human antibodies were utilized: FITC anti-human lineage cocktail including CD3 (UCHT1), CD14 (HCD14), CD16 (3G8), CD19 (HIB19), CD20 (2H7), and CD56 (HCD56). Additional lineage markers encompassing FITC anti-human CD235a (HI264), FITC anti-human FCεRIα (AER-37), FITC anti-human CD1a (HI149), FITC anti-human CD123 (6H6), and FITC anti-human CD5 (L17F12) were also added to the cocktail. APC-Cy7 anti-human CD45 (HI30), PE-Cy7 anti-human CD127 (A019D5), and PE anti-human CRTH2 (BM16) were all purchased from BioLegend, and APC anti-human NFIL3 (MABA223) was obtained from Thermo Fisher Scientific. Live/dead fixable violet or aqua cell stain kits (Thermo Fisher Scientific) were used to exclude nonviable cells, and CountBright absolute counting beads (Thermo Fisher Scientific) were applied for absolute cell number calculations. Stained cells were analyzed on a FACSCanto II system (BD Biosciences), and the data were analyzed using FlowJo version 10 software (FlowJo, LLC).

### Evaluation of lung function and examination of lung tissue structure.

Lung function was evaluated using the FinePointe RC system (Buxco Research Systems). Mice were anesthetized and mechanically ventilated according to established protocols to ensure their comfort and well-being during the assessment (55). Aerosolized PBS (baseline) and escalating doses of methacholine (Sigma) ranging from 5 to 80 mg/mL were administered to the mice. Maximal pulmonary resistance and minimal dynamic compliance were recorded during a 3-minute interval following each challenge. For histologic analysis, the right lung lobe was excised and preserved in 10% PFA. The lungs were then embedded in paraffin, and 4 mm sections were prepared for subsequent H&E staining. Composite figures were generated from the resulting images using Adobe Illustrator software (version 22.1). Histological samples were visualized using a Keyence BZ-9000 microscope and analyzed with ImageJ analysis application (NIH and Laboratory for Optical and Computational Instrumentation, University of Wisconsin).

### Examination of transcriptomic profiling data.

ILC2s were harvested and lysed in RLT buffer (Qiagen), followed by RNA extraction utilizing the MicroRNeasy kit (Qiagen). For cDNA synthesis, 10 pg of RNA from each sample was utilized as input with the SMARTer Ultra Low Input RNA v3 kit (Clontech) for library preparation. Following sample amplification, sequencing was performed on a NextSeq 500 system (Illumina), yielding an average of approximately 30 million reads per sample. The raw reads underwent processing utilizing Partek Genomics Suite software, version 7.0. Subsequently, alignment of the raw reads was accomplished using STAR 2.6.1d with the mouse reference index mm10 and GENECODE M21 annotations. Following alignment, further quantification and normalization of the reads were conducted, and differential analysis was executed employing DESeq. Gene enrichment analysis was subsequently performed utilizing the IPA tool.

### Measurement of cytokines.

Following the treatment of ILC2s with the indicated reagents, culture supernatants were collected for cytokine analysis. The LEGENDplex Mouse Th2 Panel (BioLegend) was used to assess cytokine levels in these supernatants, adhering to the manufacturer’s instructions. Similarly, cytokine measurements from BAL fluid were conducted by harvesting and analyzing the associated supernatants using the same procedures. For human cytokine analysis, culture supernatants were gathered, and cytokine levels were evaluated using the LEGENDplex Human Th2 Panel (BioLegend), following the manufacturer’s guidelines. Each LEGENDplex was measured with an Attune NxT flow cytometer (Thermo Fisher Scientific).

### Isolation and culture of hILC2s.

Peripheral hILC2s were isolated with high purity (>95%) from PBMCs of 6–10 healthy donors using the FACSAria III system, following the gating strategy shown in Supplemental Figure 10 and as previously described (7, 8, 17, 21). The process began by diluting fresh human blood at a 1:1 ratio in PBS, followed by PBMC isolation using SepMate-50 separation tubes (STEMCELL Technologies) according to the manufacturer’s instructions. After red blood cell lysis (BioLegend), CRTH2^+^ cells were isolated using the CRTH2 MicroBead Kit (Miltenyi Biotec) as per the manufacturer’s protocol. The isolated hILC2s were phenotypically characterized as CD45^+^, CD127^+^, and CRTH2^+^ cells, negative for lineage markers (CD3, CD5, CD14, CD16, CD19, CD20, CD56, CD235a, CD1a, and CD123). These ILC2s were then cultured for 72 hours at 37°C (5 × 10^4^ cells/mL) in complete RPMI supplemented with recombinant human IL-2 (10 ng/mL; BioLegend) and IL-7 (10 ng/mL; BioLegend), with or without IL-33 (100 ng/mL; BioLegend) in U-bottom 96-well plates. For specific analyses, 10 μg/mL of human CD275 (B7-H2) (MIH12: Thermo Fisher Scientific) was added to the cultures. In experiments involving PCR, the hILC2s were cultured with IL-2, IL-7, and IL-33 for 14 days, with the culture medium changed every 2–3 days to achieve the desired cell numbers.

### Quantitative reverse transcription PCR.

Total RNA was prepared using the MicroRNeasy kit (Qiagen) following the manufacturer’s instructions. The cDNA was synthesized using the iScript cDNA Synthesis Kit (Bio-Rad Laboratories). Quantitative reverse transcription PCR was then performed with specifically designed oligonucleotide primers on the CFX Connect Real-Time PCR Detection System (Bio-Rad Laboratories) as previously described (56). Amplification products were quantified using iQ SYBR Green Supermix (Bio-Rad Laboratories). Target gene expression levels were normalized to the expression of GAPDH, which served as an endogenous control. The sequences of the gene-specific primers are as follows: GAPDH forward, CAGTATGACTCCACTCACGGC, and reverse, GAGGGGCCATCCACAGTCTTC; CYP11A1 forward, TTTTTGCCCCTGTTGGATGCA, and reverse, CCCTGGCGCTCCCCAAAAAT; CYP11B1 forward, TGTGTGATGCTGCCGGAGGA, and reverse, CGCAATCGGTTGAAGCGCC; CYP17A1 forward, CCATTTCCTGAACGCACCGG, and reverse, AGAGAGGCCAAGGAAACAGGGCT; CYP21A2 forward, CGGACCTGTCCTTGGGAGACTACTCC, and reverse, CTGGGCTCTCATGCGCTCACA; and HSD3B2 forward, GCGGCTAATGGGTGGAATCTA, and reverse, CATTCTTGTTCAGGGCCTCAT.

### Statistics.

All experiments were performed 3 or 4 times independently, and data are presented as mean ± SD or SEM and analyzed utilizing GraphPad Prism software (version 9.5.1). Two-tailed Student’s *t* tests for unpaired data were employed for comparing 2 groups, and 1- or 2-way ANOVA tests were utilized for multigroup comparisons.

### Study approval.

The animal study was conducted in accordance with established protocols approved by the IACUC. The human study received approval from USC. The IRB of USC reviewed and approved the study, ensuring that it was conducted in accordance with the Declaration of Helsinki. Prior to their involvement in the study, written informed consent was obtained from all participants.

### Data availability.

The bulk RNA-Seq data have been uploaded to the Gene Expression Omnibus database (GSE288711). Additional information is available in the Supporting Data Values file.

## Author contributions

YS designed and performed experiments, analyzed data, and wrote the first draft of the manuscript. YS, KS, SS, BPH, and MHK helped perform experiments and animal husbandry for experiments. XL helped with RNA-Seq analysis and data interpretation. OA supervised the study, designed the experiments, interpreted the data, and finalized the manuscript. All authors critically read the manuscript.

## Supplementary Material

Supplemental data

Supporting data values

## Version 1 07/08/2025

In-Press Preview

## Version 2 09/16/2025

Electronic publication
