# Supplementary material for: ICOS regulates IL-10 production in group 2 innate lymphoid cells via cholesterol and cortisol biosynthesis
Source: J Clin Invest. 2025 Jul 8;135(18):e193134. doi: 10.1172/JCI193134 (PMC12435846; doi:10.1172/JCI193134)

Supplementary Figure. 1

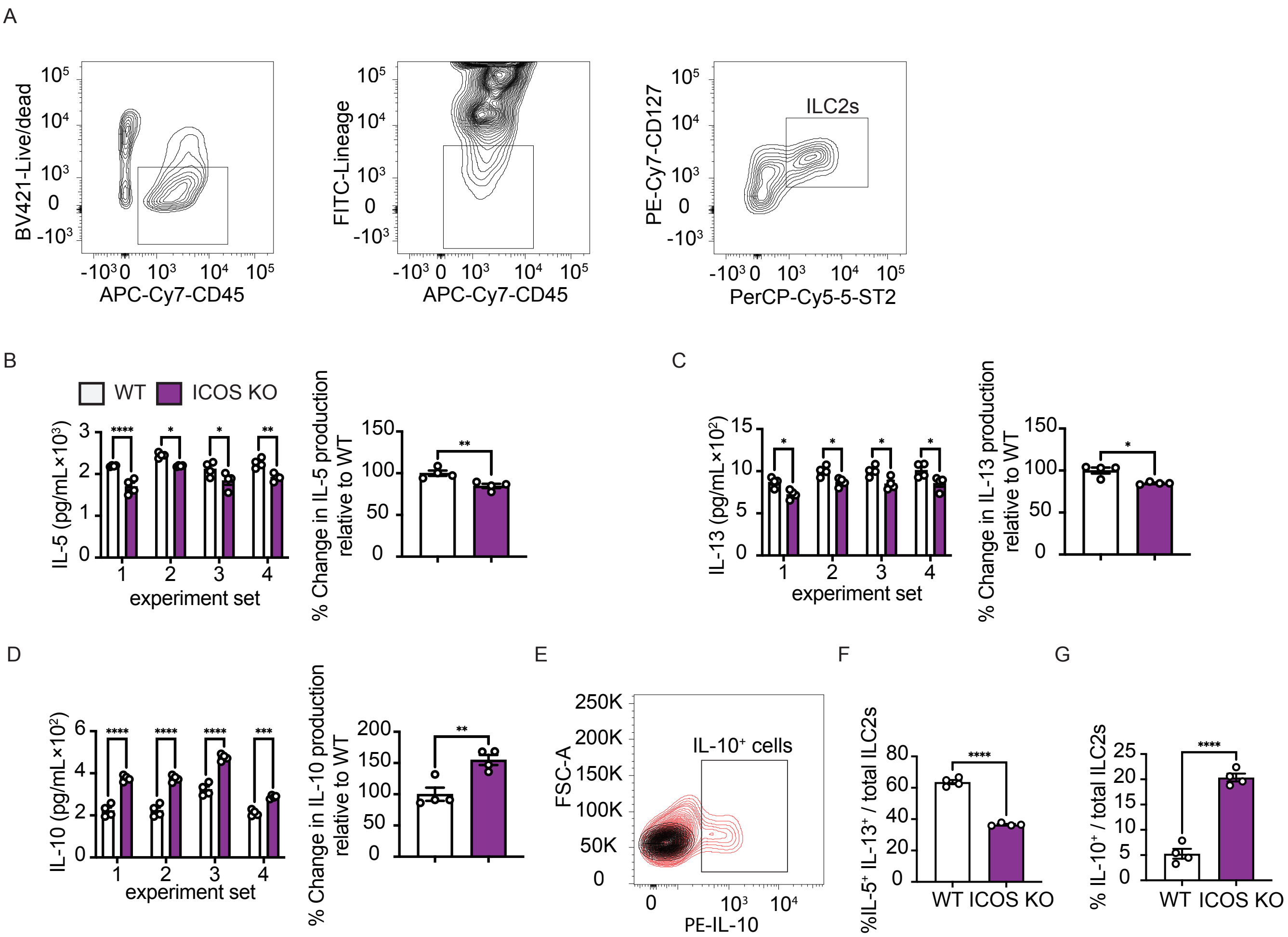

Supplementary Figure. 2

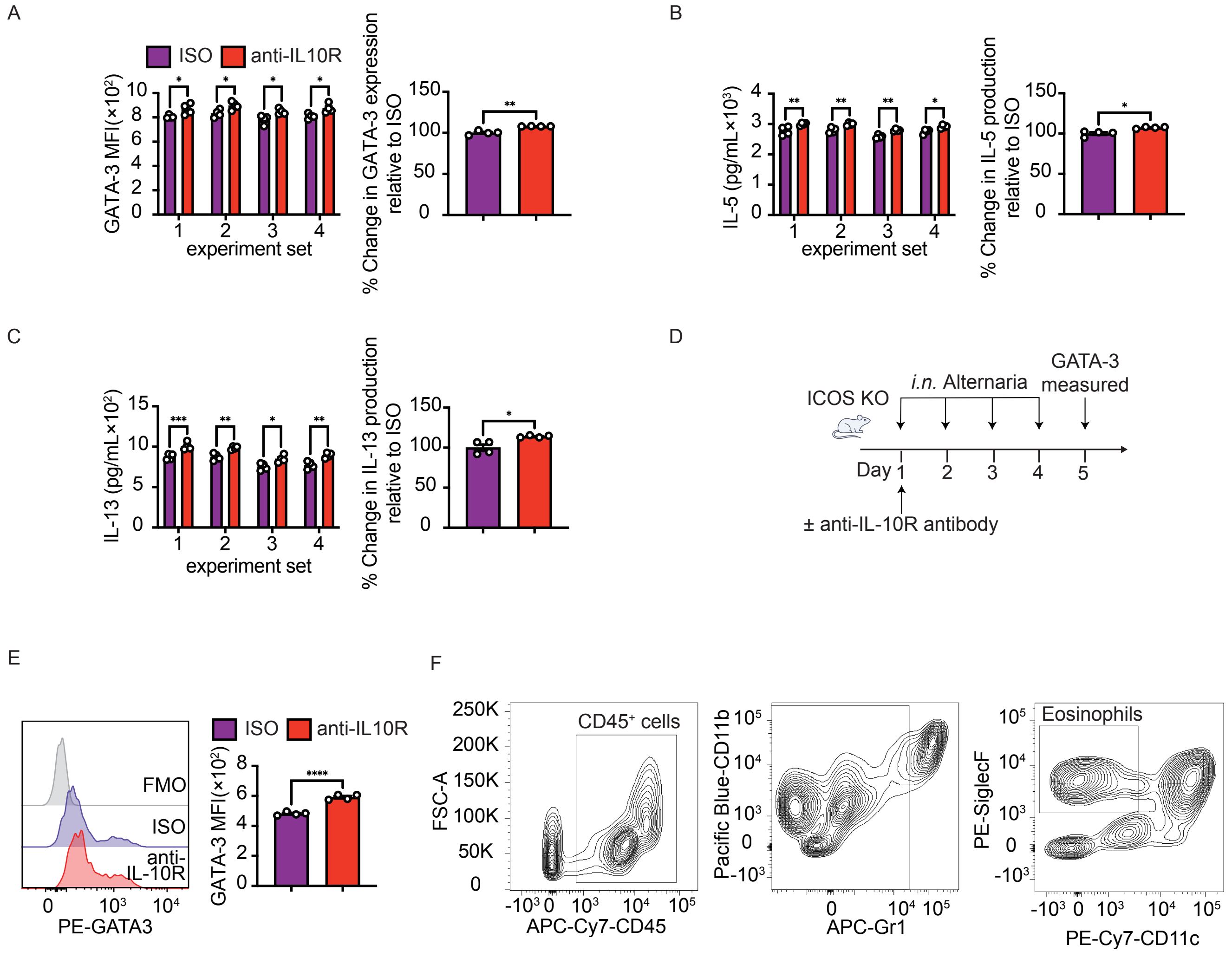

Supplementary Figure. 3

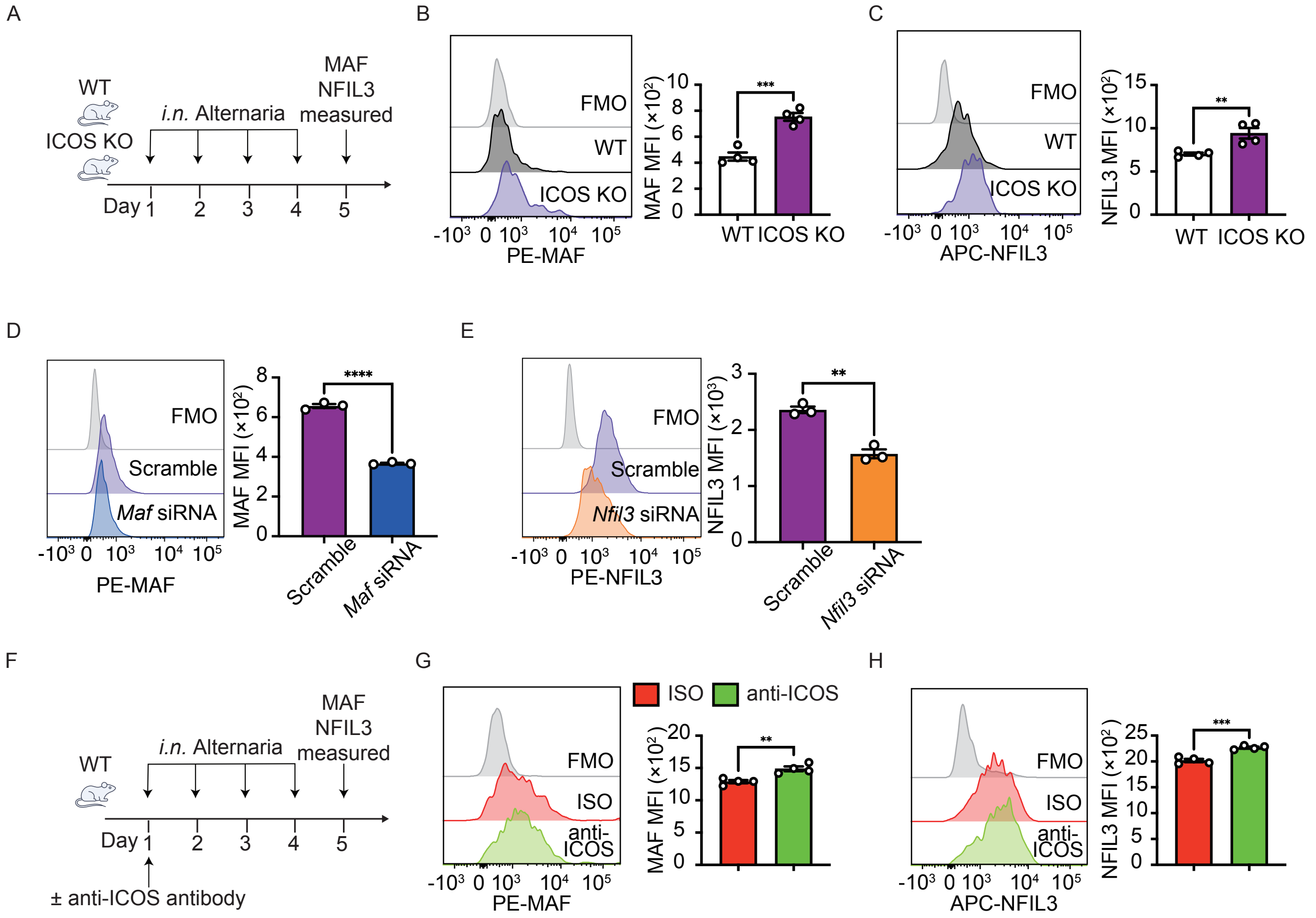

Supplementary Figure. 4

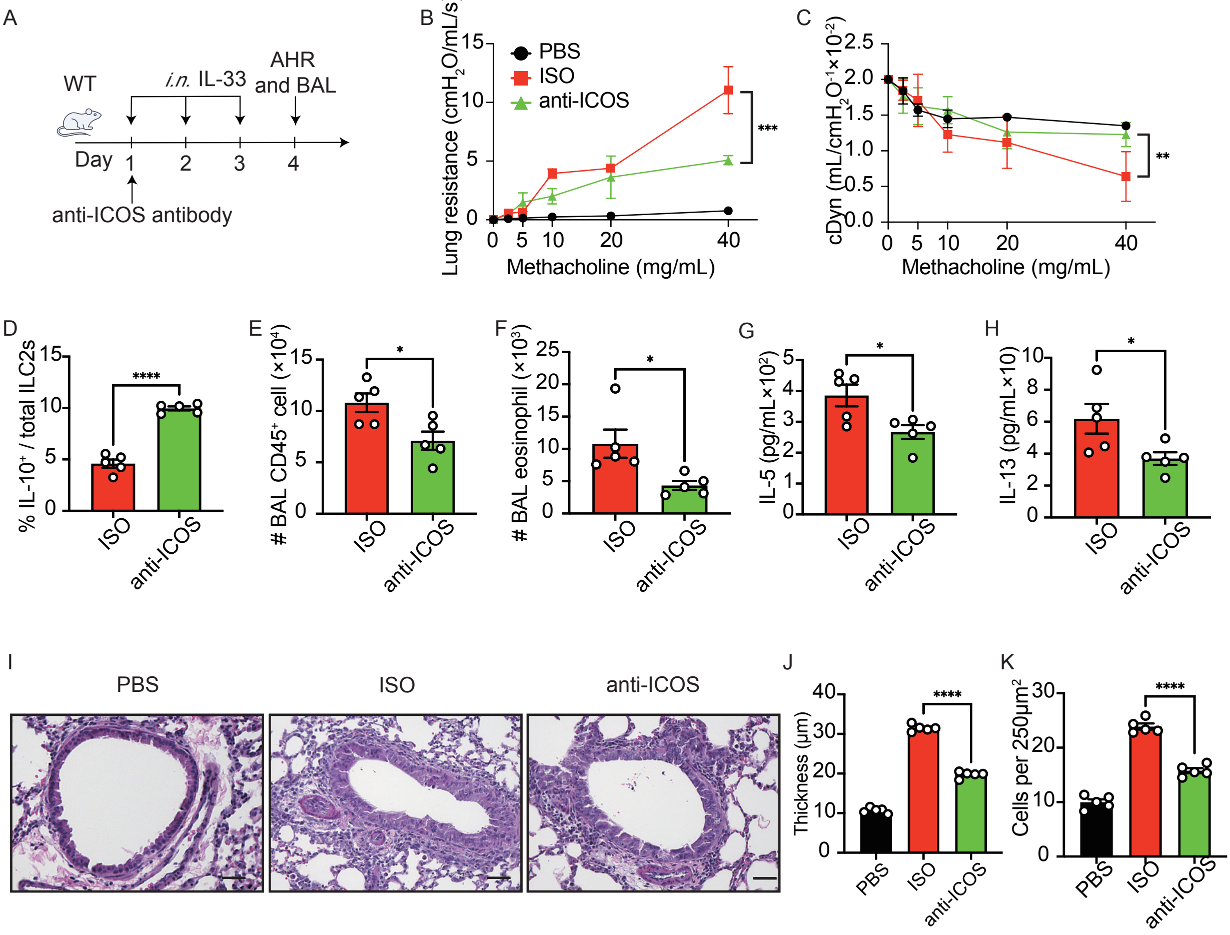

Supplementary Figure. 5

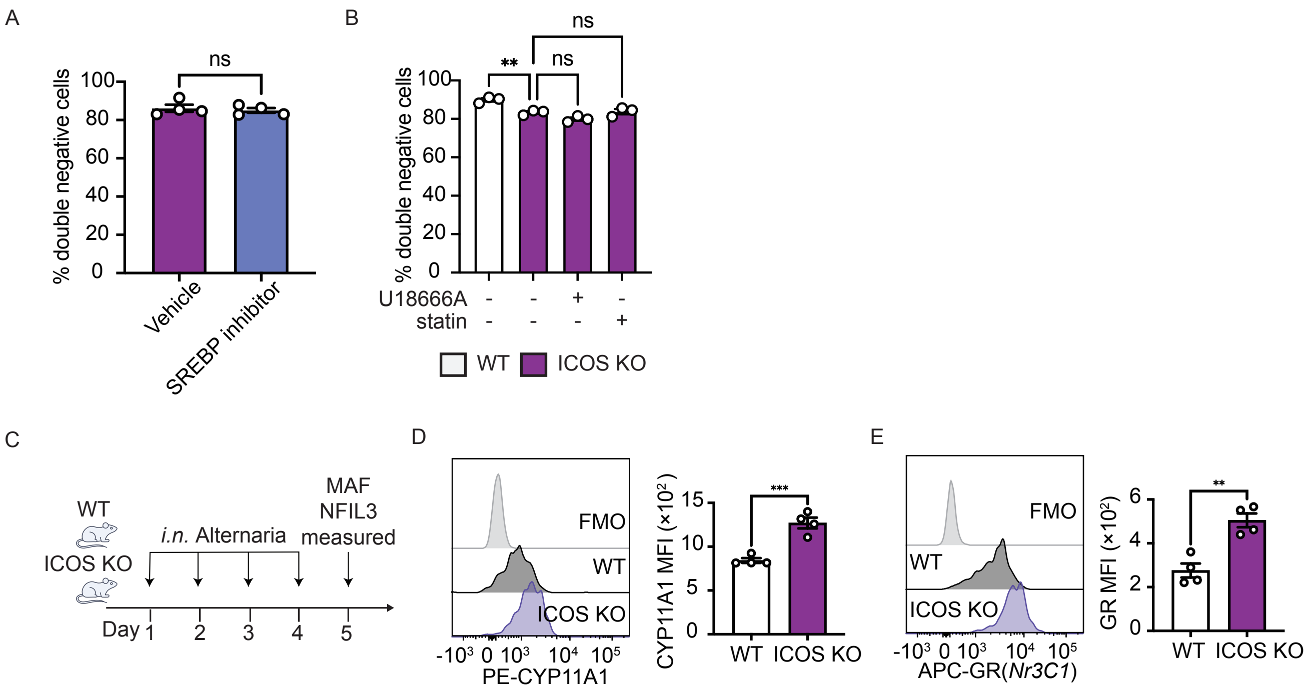

Supplementary Figure. 6

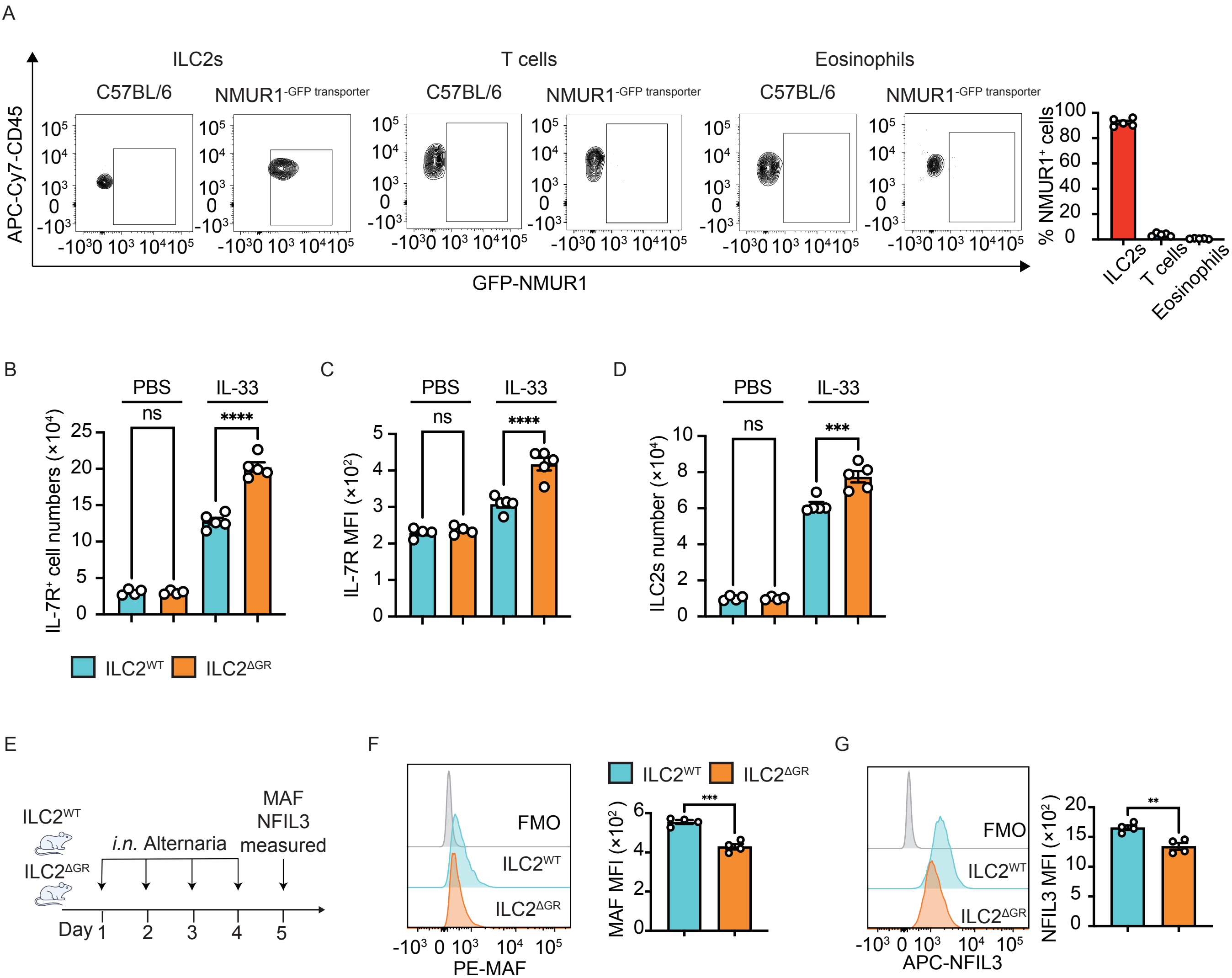

Supplementary Figure. 7

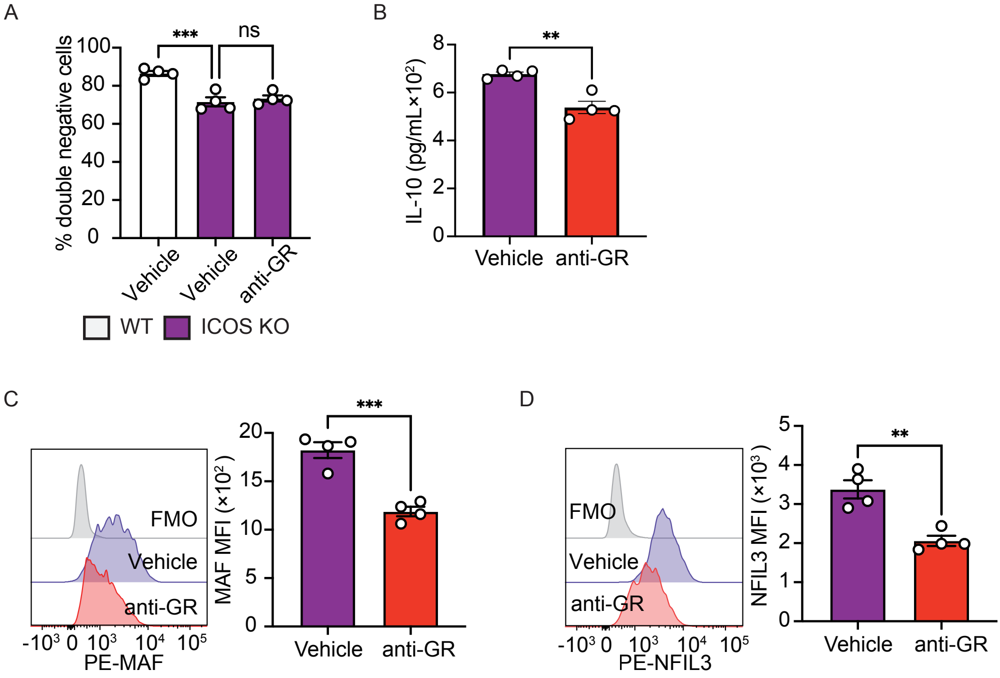

Supplementary Figure. 8

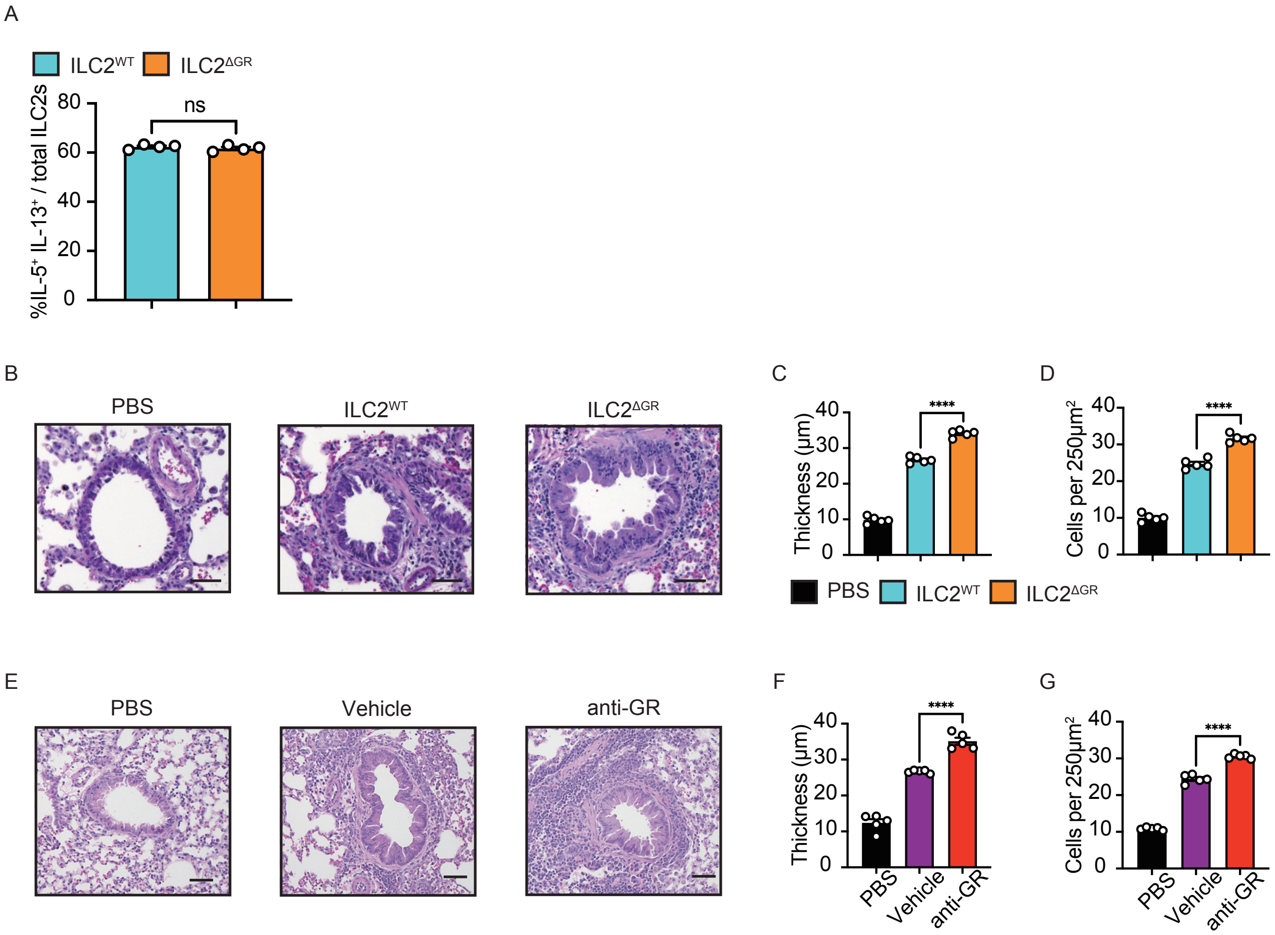

Supplementary Figure. 9

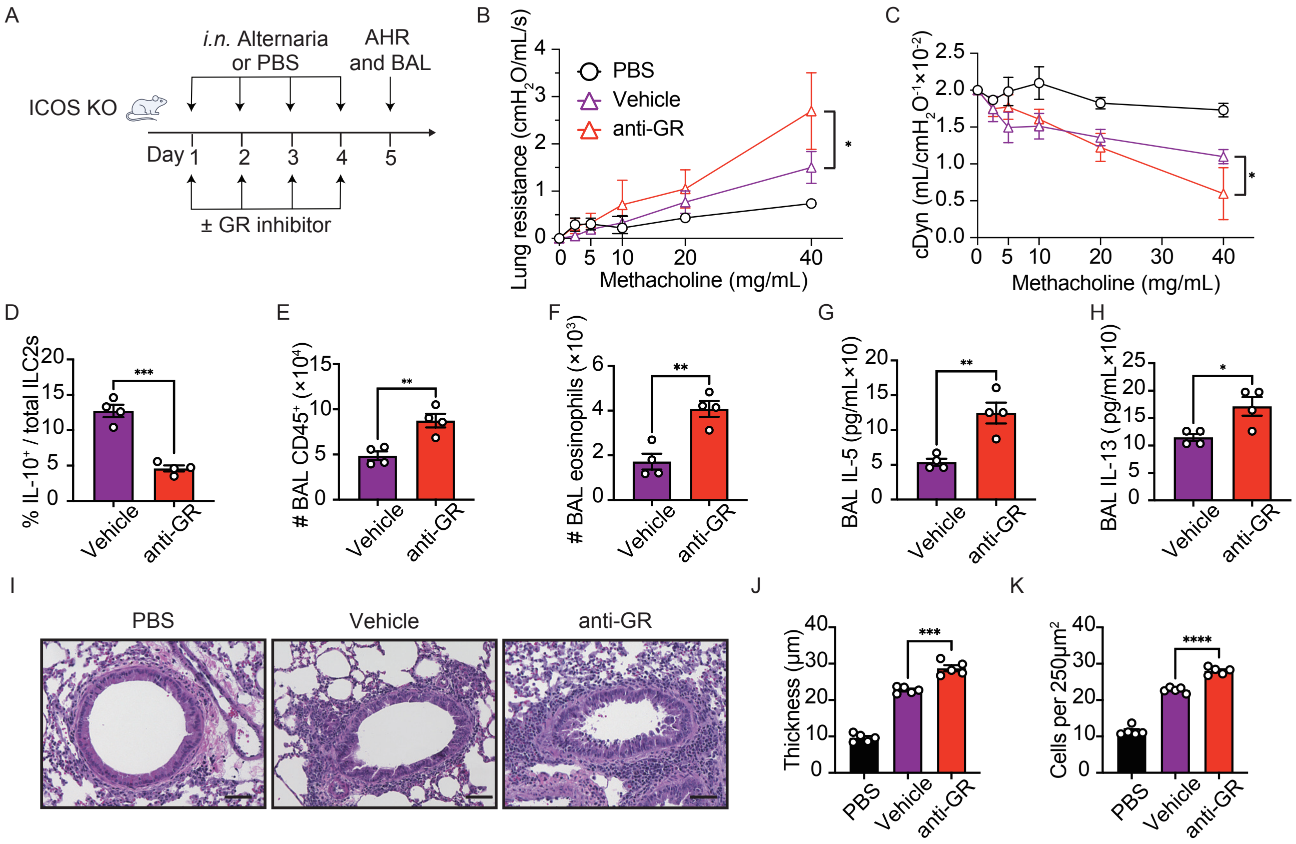

Supplementary Figure. 10

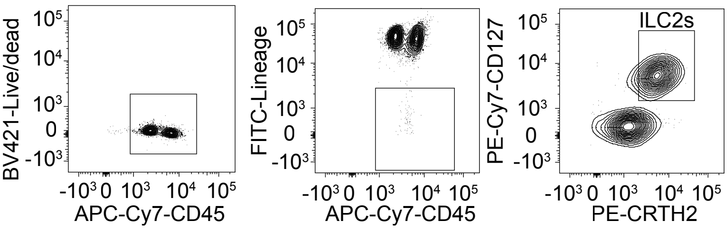

Supplement: Supplemental data [file jci-135-193134-s108.pdf]
